# MiR‐513a promotes human erythroid differentiation by modulating c‐Jun

**DOI:** 10.1002/2211-5463.70226

**Published:** 2026-03-08

**Authors:** MinJung Kim, Brittany Taylor, Shannon Bolten, Christian L. Eberly, Mahliya Abdurahman, T. Michael Creed, Acong Yang, Taylor L. Hatchet, Tristan Dyson, Shuo Gu, Curt I. Civin, Tami J. Kingsbury

**Affiliations:** ^1^ Center for Stem Cell Biology & Regenerative Medicine University of Maryland School of Medicine Baltimore MD USA; ^2^ Department of Pediatrics University of Maryland School of Medicine Baltimore MD USA; ^3^ Department of Physiology University of Maryland School of Medicine Baltimore MD USA; ^4^ RNA Biology Laboratory, Center for Cancer Research, National Cancer Institute Frederick MD USA

**Keywords:** c‐JUN, erythroid differentiation, erythropoiesis, GATA1, microRNAs

## Abstract

Erythropoiesis is a highly coordinated process that generates mature red blood cells from hematopoietic stem‐progenitor cells (HSPCs). Erythropoietin (EPO) is a major regulator of erythropoiesis and binds to the erythropoietin receptor (EPOR), leading to increased erythroid differentiation and proliferation. MicroRNAs (miRs) are important developmental regulators, and distinct miR expression patterns are associated with specific stages of hematopoietic differentiation. Expression profiling of EPO‐stimulated human HSPCs revealed increased expression of miR‐513a‐5p in early erythroid cells. Enforced expression of miR‐513a in primary human CD34^+^ HSPCs promoted erythroid differentiation, as determined by cell‐surface marker expression and increased levels of erythroid molecules including hemoglobin. Similar results were observed in human TF‐1 erythroleukemia cells, where miR‐513a stimulated erythroid differentiation including increased GATA1 and hemoglobin expression and decreased GATA2 expression, even in the absence of EPO. Notably, miR‐513a promoted erythroid differentiation in EPOR knockout (KO) TF‐1 cells, but not in GATA1 KO TF‐1 cells, indicating that miR‐513a requires GATA1, but not EPOR, to stimulate erythropoiesis. Further analysis revealed that enforced expression of miR‐513a was associated with reduced c‐Jun and phospho‐c‐Jun protein levels. Overexpression of c‐Jun inhibited both EPO‐ and miR‐513a‐stimulated erythropoiesis. Conversely, c‐Jun KO TF‐1 cells had increased hemoglobin protein expression, even in the absence of EPO, phenocopying miR‐513a overexpression. In summary, this study identifies miR‐513a as a positive regulator of early human erythropoiesis and supports a role for miR‐513a in promoting erythroid differentiation by modulating c‐Jun expression and increasing GATA1 expression.

## Graphical abstract

During early human erythropoiesis, miR‐513a promoted erythroid differentiation in primary human CD34^+^ hematopoietic stem‐progenitor cells and human TF‐1 erythroleukemic cells by indirectly decreasing c‐Jun and phospho‐c‐Jun expression, which are associated with increased GATA1 expression. Notably, miR‐513a promoted erythroid differentiation in EPOR‐knockout‐TF‐1 cells, but not in GATA1‐Knockout‐TF‐1 cells, indicating that miR‐513a requires GATA1, but not EPOR, to stimulate erythropoiesis.

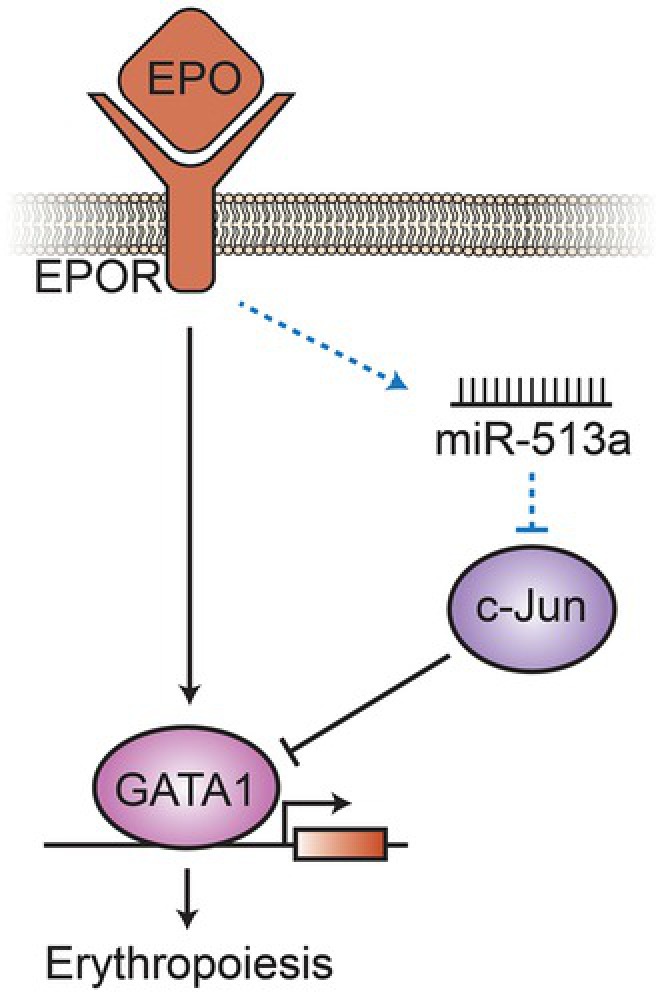

**Abbreviations**
7‐AAD: 7‐Aminoactinomycin D
ALAS2: Delta‐aminolevulinate synthase 2
BrdU: Bromodeoxyruidine
CRISPR: Clustered regularly interspaced short palindromic repeats
EF1α: human elongation factor‐1 alpha
FACS: fluorescence‐activated cell sorting
GAPDH: Glyceraldyhyde‐3‐phophate dehydrogenase
GATA1: GATA‐binding protein 1
GATA2: GATA‐binding protein 2
GFP: green fluorescent protein
HBA: Alpha hemoglobin
HBB: Beta hemoglobin
HBG: Gamma hemoglobin
hUbC: human ubiquitin C
SLC4a1: Solute carrier family 4 – anion exchanger – member 1

Erythropoiesis, the process by which rare hematopoietic stem‐progenitor cells (HSPCs) generate mature red blood cells, is regulated by multiple transcriptional and post‐transcriptional mechanisms that control cell lineage commitment/fate, proliferation/survival, and differentiation/maturation [1, 2]. Since mature red blood cells are relatively short‐lived, the average adult human generates 2–3 million cells per second under normal physiologic conditions, and several fold higher during conditions of erythroid stress [1, 2]. Erythropoiesis thus serves as a development paradigm in which erythroid progenitor cell differentiation occurs in conjunction with cell proliferation [3, 4]. Erythropoietin (EPO) is a major regulator of erythropoiesis and promotes erythroid differentiation/maturation and proliferation/survival of erythroid cells by binding to the erythropoietin receptor (EPOR) [3, 4]. During erythropoiesis, EPO signaling stimulates the expression of GATA1, a major transcription factor of erythropoiesis. Conversely, the expression of GATA2, which mediates HSPC generation and maintenance, declines [5, 6]. Replacement of GATA2 at the transcriptional regulatory domains of erythroid genes by GATA1, referred to as the ‘GATA switch’, is a hallmark of erythroid commitment and a key regulatory event in erythropoiesis [7, 8, 9, 10].

MicroRNAs (miRs), small (~22 nucleotide) noncoding RNAs, regulate numerous processes, including differentiation, proliferation, and apoptosis, by multiple mechanisms including translational inhibition, post‐translational degradation, and transcriptional stimulation of target mRNAs [11]. In recent years, miRs have emerged as important regulators of erythropoiesis by regulating erythroid‐specific gene expressions, as well as key events such as GATA switching and hemoglobin switching [11, 12, 13]. Expression profiling of hematopoietic cell subpopulations has revealed differential miR expression at specific hematopoietic stages and lineages [11, 14, 15]. For example, miR‐451 and miR‐210 are upregulated and miR‐221 and miR‐222 are downregulated in mouse and human erythroid cells [11, 16, 17, 18]. However, most studies have focused on miRs in late‐stage erythroid cells following long‐term erythroid differentiation culture [19, 20, 21, 22, 23, 24]. Therefore, we profiled miR expression in immunopurified early erythroid progenitors generated from peripheral blood‐derived CD34^+^ HSPCs cultured with EPO for a short‐term period to gain insight into miRs regulating early human erythropoiesis. Among upregulated miRs that could have a role in promoting erythropoiesis, we focused on miR‐513a‐5p because it has not been previously studied in erythropoiesis. Here we report that miR‐513a‐5p is upregulated in early erythroid cells and that enforced miR‐513a expression promotes human erythroid differentiation. Furthermore, our findings indicate that miR‐513a‐mediated erythroid differentiation is associated with reduced c‐Jun expression and requires GATA1.

## Materials and methods

### MiR expression profiling

**Fig. 1:**
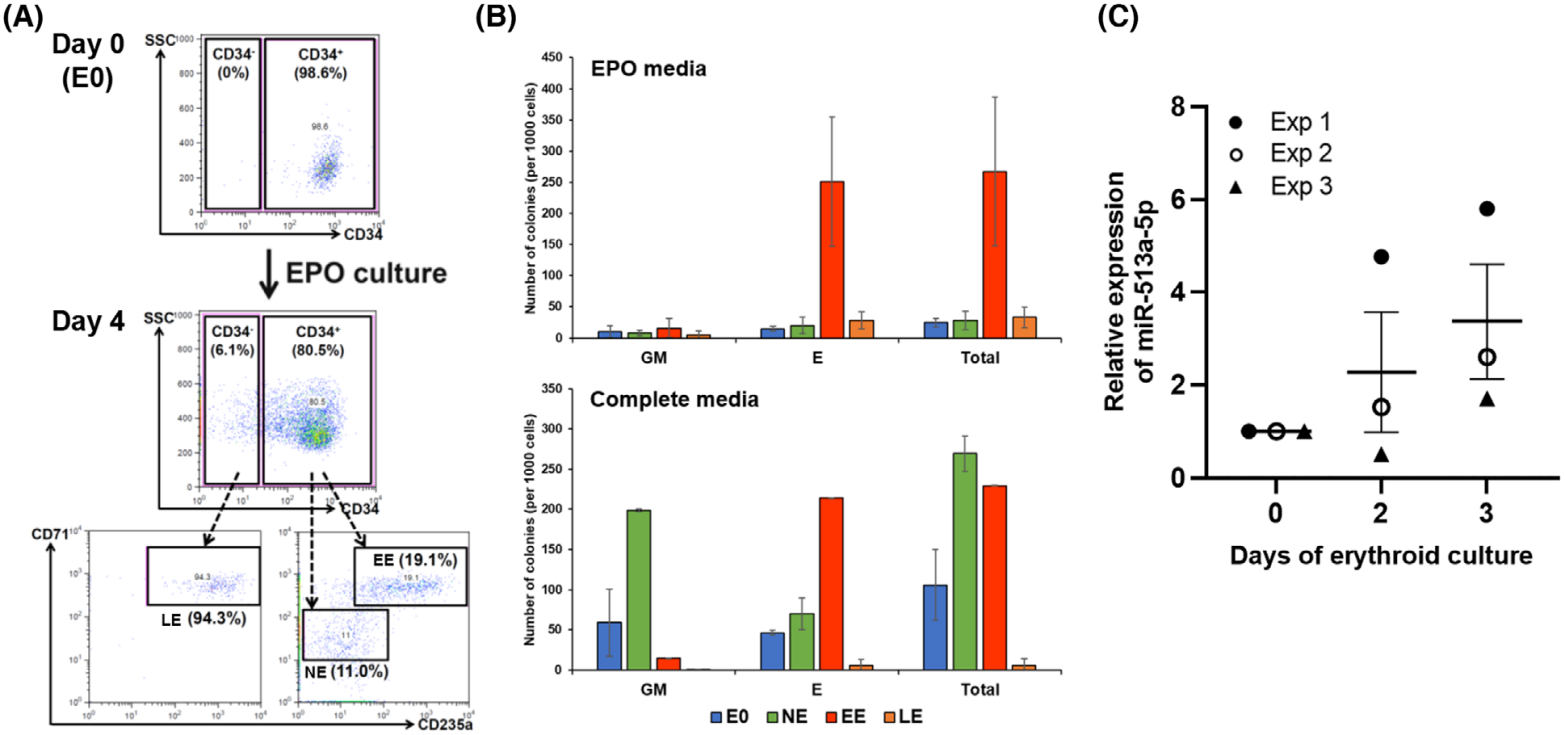
MiR‐513a‐5p expression increases during early EPO‐stimulated erythroid differentiation. (A) Primary human CD34^+^ HSPCs (E0; uncultured HSPCs) were cultured in EPO‐containing media. After 4 days of erythroid culture, differentiated cells were stained with anti‐CD71, CD235a, and CD34 antibodies, and FACS‐sorted into non‐erythroid cells (NE; CD34^hi^CD71^low^CD235a^low^), early erythroid cells (EE; CD34^hi^CD71^hi^CD235a^hi^), and late erythroid cells (LE; CD34^low^CD71^hi^CD235a^hi^) cell populations. (B) FACS‐sorted cells were plated in methylcellulose media containing SCF and EPO (EPO media) or the cytokine cocktail (Complete media). After 14 days of culture, erythroid colonies (E) and granulocytic‐monocytic colonies (GM) were counted (Mean ± SEM, *n* = 3). (C) Total RNAs were isolated from unsorted HSPCs cultured in EPO‐containing media on day 0, 2, or 3. MiR‐513a‐5p levels were determined by qRT‐PCR. U6 small nuclear RNA expression was used as an endogenous control. The relative expression of miR‐513a‐5p on days 2 and 3 was compared to day 0. The scatter plot is from three independent experiments and solid lines indicate the average miR‐513a‐5p expression (Mean ± SEM, *n* = 3).

Human primary CD34^+^ HSPCs were purchased from the Cellular Therapy and Cell Processing Facilities (Fred Hutchinson Cancer Center, Seattle, WA, USA). For expression profiling, HSPCs were cultured in StemSpan SFEM media (STEMCELL Technologies, Vancouver, BC, Canada) containing 100 ng·mL human stem cell factor (SCF; PeproTech, now part of Thermo Fisher Scientific, Cranbury, NJ, USA), 20 ng·mL thrombopoietin (TPO; PeproTech), 100 ng·mL FMS‐like tyrosine kinase receptor 3 ligand (FLT3L; PeproTech), and 0.25 U·mL erythropoietin (EPO; R&D Systems, Minneapolis, MN, USA) for 4 days, then resulting cells were sorted into three populations: nonerythroid (NE; CD34^hi^CD71^low^CD235a^low^), early erythroid (EE; CD34^hi^CD71^hi^CD235a^hi^) and late erythroid (LE; CD34^low^CD71^hi^CD235a^hi^), using fluorescence‐activated cell sorting (FACS) Aria (BD Bioscience, Franklin Lakes, NJ, USA; Fig. 1A). Total RNA was isolated from each sorted population, as well as from uncultured HSPCs (E0), and global miR and mRNA expressions were assayed by Cogenics (now Beckman Coulter Genomics, Indianapolis, IN, USA) using Agilent microarray technology (Agilent Technologies, Santa Clara, CA, USA). Two independent miR expression profiling experiments were performed. Microarray data was analyzed using the GeneSpring software (Agilent Technologies).

### Lentivector construction for overexpression and knockdown/knockout

To overexpress miR‐513a, the genomic DNA region harboring miR‐513a‐1 (455 bp) was amplified by PCR, and then cloned into the empty lentivector pWCC52 driving green fluorescent protein (GFP) and miR‐513a expression from a human elongation factor‐1 alpha (EF1α) promoter. This miR‐513a wild‐type (WT) lentivector was mutated using QuikChange Lightning Site‐Directed Mutagenesis kits (Agilent Technologies) to generate miR‐513a‐5p^mut^ and miR‐513a‐3p^mut^ lentivectors.

To overexpress human c‐JUN, the human c‐JUN open reading frame (ORF; 996 bp) was amplified by PCR, and then cloned into the empty lentivector pWCC43 driving c‐JUN ORF expression from an EF1α promoter and GFP expression from the human ubiquitin C (hUbC) promoter.

For CRISPR/Cas9‐mediated EPOR KO and c‐JUN KO, sgRNAs were synthesized as oligonucleotides (Integrated DNA Technologies, Coralville, IA, USA) and then annealed sgRNAs were cloned into plentiCRISPRv2GFP (a gift from David Feldser, University of Pennsylvania, Philadelphia, PA, USA; Addgene #82416).

All primers used for generating OE/KO lentivectors are listed in Table S1.

Because the lentiviral infection using different types of lentivector could cause the variation of erythroid differentiation, all OE or KO/KD experiments include their own control lentivector(s) to compare the effect of the genes/miRs of interest.

### Lentivirus production

Lentivirus production was done using a second‐generation lentiviral system. Briefly, HEK293T cells (RRID:CVCL_0063, American Type Culture Collection CRL‐3216, Manassas, VA, USA) were cotransfected with lentivector plasmids generated for this study, a packaging plasmid psPAX2 (Addgene #12260, Watertown, MA, USA) and an envelope plasmid pMD2.G (Addgene #12259) using polyethylenimine (PEI; Sigma‐Aldrich, St. Louis, MO, USA). After 24 h post‐transfection, transfected cells were cultured in low‐serum media containing DMEM (Cellgro, now Corning, Corning, NY, USA), 1% fetal bovine serum (FBS; Gemini Bio Products, West Sacramento, CA, USA) and 1X Glutamine (Thermo Fisher Scientific, Waltham, MA, USA) for an additional 48 h. Then, the media containing lentiviruses was harvested and concentrated using ultracentrifugation or PEG‐it Virus Precipitation Solution (System Biosciences, Palo Alto, CA, USA). Concentrated lentiviruses were titrated in HEK293T cells or TF‐1 cells with 8 ng·mL polybrene (Sigma‐Aldrich), and then the transduction units were calculated by measuring %GFP^+^ cells using flow cytometry analysis after 3 days post‐transduction. All experiments were performed with mycoplasma‐free cells.

### Lentivirus transduction and erythroid culture of human primary CD34 ^+^ HSPCs

As previously described [25], lentiviral transduction of HSPCs was conducted with 1 mg·mL Kolliphor P407 (Sigma‐Aldrich) and 10um 16,16‐dimethyl prostaglandin E2 (Abcam, Waltham, MA, USA), and subsequent erythroid culture of transduced HSPCs was performed in StemSpanSFEM media containing 100 ng·mL SCF and 1 U·mL EPO (TONBO Biosciences, now Cytek Biosciences, Fremont, CA, USA) in order to stimulate erythropoiesis predominantly. At indicated time points, viable cells were counted by trypan blue dye exclusion method, and erythroid differentiation was assessed by flow cytometry for correlated cell surface marker expression of CD71, CD235a, CD34, and CD105 within transduced GFP^+^ cell populations (Table S2) [26, 27] and by qRT‐PCR for mRNA expression of a panel of erythroid molecules (listed in Table S3).

### Colony‐forming cell (CFC) assay

The colony‐forming cell assay was performed according to the manufacturer's instructions (STEMCELL Technologies). Cells were plated at 1000 cells/ml in methylcellulose media containing 100 ng·mL SCF and 1 U·mL EPO (EPO media) or the cytokine cocktail (Complete media), and colonies were counted after 14 days of culture. Red colonies were counted as erythroid colonies (E) and white colonies were counted as granulocytic‐monocytic colonies (GM) based on their color and morphology. Statistical significance was determined by Student's *t*‐test.

### Lentivirus transduction and erythroid culture of TF‐1 human erythroleukemia cells

Cytokine‐dependent TF‐1 cells (RRID: CVCL_0559, American Type Culture Collection CRL‐2003) were cultured routinely in RPMI1640 media (Cellgro) containing 10% FBS and 1 ng·mL recombinant human granulocyte‐macrophage colony‐stimulating factor (GM‐CSF; TONBO Biosciences). Lentiviral transduction was conducted with 8 ng·mL polybrene, and subsequent erythroid culture of transduced TF‐1 cells was performed in RPMI1640 media containing 5% FBS and 1 U·mL EPO [25, 28]. Analysis of erythroid cells at indicated time points was performed as described above for flow cytometry and qRT‐PCR, and as previously described for western blot (Table S4) [25, 28]. All experiments were performed with mycoplasma‐free cells.

### Flow cytometry analysis

Erythroid differentiation was quantified by multicolor flow cytometry analysis of CD71, CD235a, CD34, or CD105 surface expression after immunostaining with monoclonal antibodies (Antibodies are listed in Table S2; an example of the FACS gating is shown in Fig. S1). For erythroid differentiation, transduced cells were stained with anti‐CD71, CD235a, CD34, or CD105 monoclonal antibodies, followed by flow cytometry analysis using an Aurora spectral flow cytometer (Cytek Biosciences) or an Accuri C6 flow cytometer (BD Bioscience). For GFP competition assays, transduced TF‐1 cells (at MOI = 0.5; ~50% GFP^+^ cells on Day 3 post‐transduction) were assessed weekly by flow cytometry analysis to determine the percentage of GFP^+^ cells. The BrdU/7‐AAD staining was performed according to the manufacturer's instructions (APC BrdU Kit, BD Bioscience). For the AnnexinV/7‐AAD staining, transduced TF‐1 cells were resuspended in 1X AnnexinV Binding Buffer (BD Bioscience), followed by staining with AnnexinV‐APC antibody (BioLegend, San Diego, CA, USA) and 7‐AAD (BioLegend). Flow cytometry analysis of GFP competition assays, BrdU/7‐AAD staining, or AnnexinV/7‐AAD staining was performed using the Accuri C6 flow cytometer (BD Bioscience). All flow cytometry data were analyzed using FlowJo software (BD Bioscience), and statistical significance was determined by Student's *t*‐test.

### RNA isolation and qRT‐PCR

Total RNA was isolated using the miRNeasy Mini Kit (Qiagen, Germantown, MD, USA) and quantified using a NanoDrop ND‐1000 spectrophotometer (Thermo Fisher Scientific). To examine the expression of miR‐513a‐5p, cDNAs were synthesized from 100 to 200 ng of total RNA using the Mir‐X miRNA First‐Strand Synthesis Kit (Clontech, Mountain View, CA, USA) and qPCR was performed with TB Green Advantage qPCR Premix (Clontech). U6 small nuclear RNA (U6 snRNA) was used as an endogenous control for normalization of miR Ct values according to the manufacturer's instructions (Fig. S2). For miRNA analysis, miR‐513a‐5p expression was first normalized to U6 snRNA expression. Relative miR‐513a‐5p expression levels at Days 2 and 3 were then calculated by normalization to the corresponding miR expression level at Day 0. To assess mRNA expression of erythroid‐specific molecules including hemoglobins (HBB, HBG, HBA), GATA1, GATA2, ALAS2, and SLC4a1, cDNAs were synthesized from 0.5 to 2 μg total RNA using the High Capacity RNA‐to‐cDNA kit (Thermo Fisher Scientific) and qPCR was performed with Power SYBR Green PCR Master Mix (Applied Biosystems, Carlsbad, CA, USA). GAPDH or β‐Actin was used as an endogenous control to normalize Ct values. For mRNA analysis, expression levels of erythroid‐specific genes were normalized to GAPDH mRNA expression. Relative mRNA expression levels in miR‐513a OE‐HSPCs or miR‐513a OE‐TF‐1 cells were then calculated by normalization to the corresponding mRNA expression levels in control empty vector (EV)‐transduced HSPCs or EV‐TF‐1 cells, respectively. All qRT‐PCR were performed and analyzed using QuantStudio 6 Flex Real‐Time PCR Software (Thermo Fisher Scientific) according to the manufacturer's instructions, and statistical significance was determined by Student's *t*‐test. Primer sequences used for qRT‐PCR are listed in Table S3.

### Western blots

Protein whole cell lysates were prepared in lysis buffer [radioimmunoprecipitation (RIPA; Sigma‐Aldrich) buffer containing 1 mm phenylmethanesulfonyl fluoride (Sigma‐Aldrich) and 1 tablet of complete protease inhibitor cocktail (Roche, Indianapolis, IN, USA)]. Protein concentration was determined using Bio‐Rad Protein assay reagents (Bio‐Rad Laboratories, Hercules, CA, USA) according to the manufacturer's instructions. Five to twenty micrograms of protein whole cell lysates were separated on a pre‐made 4–12% Bis‐Tris NuPAGE gel (Thermo Fisher Scientific) and transferred to a polyvinylidene difluoride (PVDF) membrane using an iBlot Dry Blotting system (Thermo Fisher Scientific). Membranes were incubated with HBB, HBG, HBA, GATA1, GATA2, EPOR, c‐JUN, or phospho‐c‐JUN antibodies, and then reprobed with β‐actin or α‐tubulin antibody as a loading control. Signal was detected using Supersignal West Pico PLUS Chemiluminescent Substrate kit (Thermo Fisher Scientific) with a ChemiDOC XRS+ system (Bio‐Rad Laboratoreis) and quantified using IMAGE LAB software (Bio‐Rad Laboratories). For western blot analysis, target protein signals were first normalized to the corresponding β‐actin or α‐tubulin signal in each lane. Relative protein expression levels in TF‐1 cells transduced with miR‐513a OE, EPOR KO, GATA1 KO, c‐JUN OE, or c‐JUN KO lentivirus were subsequently normalized to those in the corresponding empty vector control lentivirus‐transduced TF‐1 cells. Statistical significance was determined by Student's *t*‐test. All antibodies used for the western blot analysis are listed in Table S4.

## Results

### MiR‐513a‐5p expression increases during early erythroid differentiation of HSPCs

To identify miRs that function in early stages of human erythropoiesis, we profiled miR expression in uncultured HSPCs (E0; > 98% CD34^hi^CD71^low^CD235a^low^) versus early erythroid progenitors (EE; CD34^hi^CD71^hi^CD235a^hi^) (Fig. 1A). To confirm early erythroid cells were enriched within the EE population, colony forming cell (CFC) assays were conducted in EPO and Complete methylcellulose media (Fig. 1B). In EPO media, which supports erythroid colonies, the erythroid colony‐forming cell frequency was predominantly observed in EE, as compared to NE, LE, or uncultured HSPCs (E0). In Complete media, which supports multiple myeloid lineages, the erythroid colony‐forming cells were enriched in EE, whereas the granulocytic‐monocytic (GM) colony‐forming frequency was higher in NE. As expected, CD34^low^ LE population exhibited a very low colony‐forming frequency. These observations are consistent with a previous publication [29, 30] demonstrating that early committed erythroid progenitors were highly enriched in the EE population. To identify miRs that are differentially expressed between uncultured HSPCs (E0) and early erythroid cells (EE), total RNAs from E0 and EE populations were subjected to miR microarray profiling. We identified 40 differentially expressed miRs in the EE population as compared to the E0 population, including 12 upregulated and 28 downregulated miRs (cutoff >5‐fold change; Fig. S3 and Table S5). Of these, 7 upregulated miRs (including miR‐144, miR‐144*, miR‐451, miR486‐3p, miR‐486‐5p, mR‐210 and miR‐18b) and 13 downregulated miRs (including miR‐150, miR‐146a, miR‐125b, miR‐181c, miR‐145, miR‐125‐5p, miR‐221*, miR‐363, miR‐26a, miR‐27a, miR‐222, let‐7e and miR‐376a) have been implicated in normal or impaired erythropoiesis (Fig. S3). Among upregulated miRs that could have a role in promoting erythropoiesis, we focused on miR‐513a‐5p, which has not been studied previously in erythropoiesis. We confirmed by qRT‐PCR that endogenous miR‐513a‐5p levels increased during the early stages of EPO‐stimulated erythroid cultures (Fig. 1C), suggesting a potential role for miR‐513a‐5p in early human erythropoiesis.

### MiR‐513a promotes erythroid differentiation in HSPCs

**Fig. 2:**
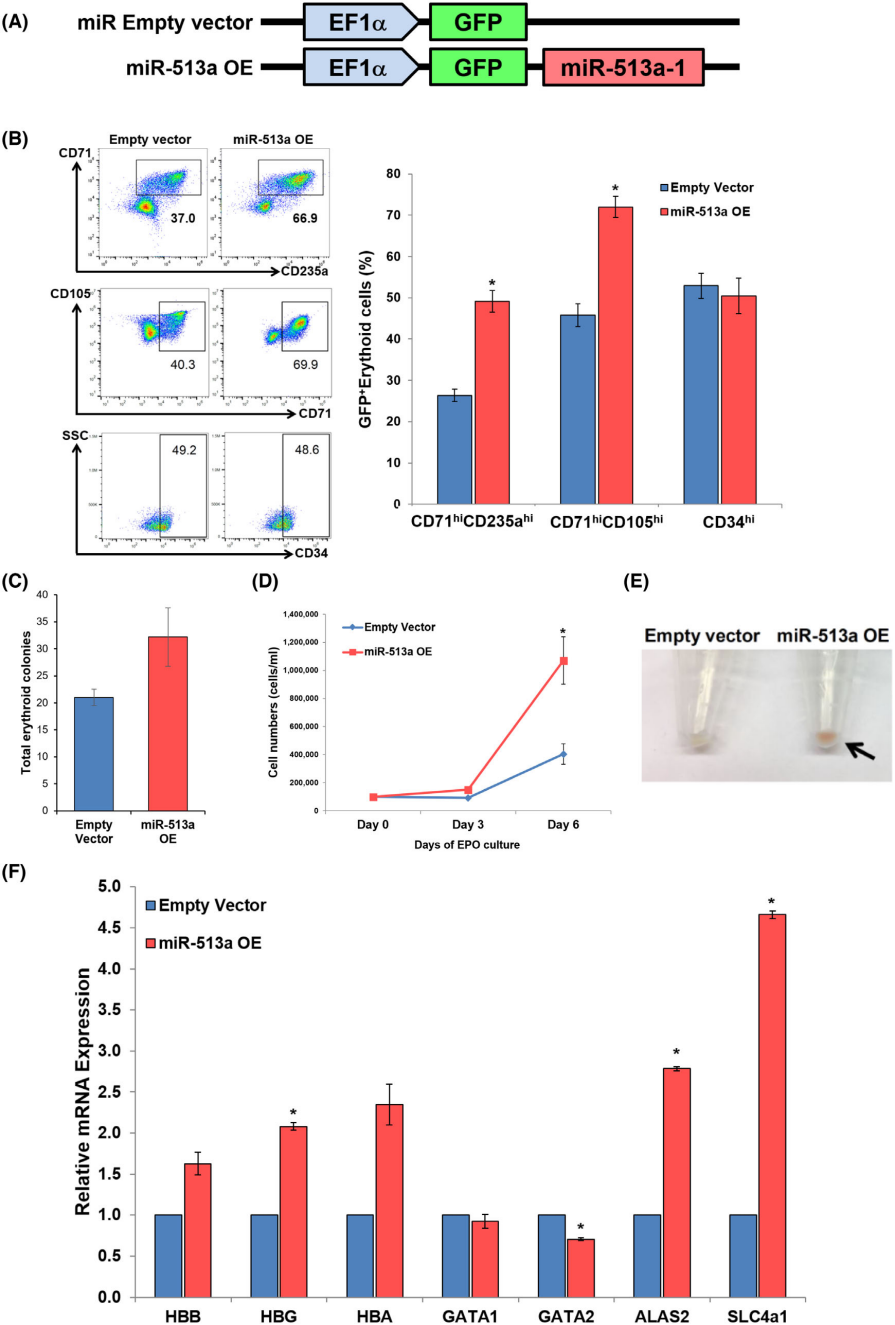
Enforced miR‐513a expression promotes erythroid differentiation in primary human HSPCs. (A) Schematic diagram of lentivectors used for miR‐513a overexpression experiments. (B–F) HSPCs were transduced with the miR empty vector or miR‐513a OE lentivirus for 3 days. Transduced HSPCs were cultured in media containing SCF and EPO for either 3 or 6 days prior to analysis or plated in methylcellulose media for the CFC assays. (B) On day 3 of erythroid culture, HSPCs were immunostained with anti‐CD71, CD235a, CD105, and CD34 antibodies. FACS plots are from a representative experiment. The numbers in the plots indicate %CD71^hi^CD235a^hi^ (top), %CD71^hi^CD105^hi^ (middle), or %CD34^hi^ (bottom) in the GFP^+^ cell population. Bar graphs indicate the average of %CD71^hi^CD235a^hi^, %CD71^hi^CD105^hi^, or %CD34^hi^, respectively, from three independent experiments (Mean ± SEM, *n* = 3). Statistical significance was determined by Student's *t*‐test (*P < 0.05). (C) Transduced HSPCs were plated in methylcellulose media containing SCF and EPO for the CFC assays. Erythroid colonies were counted after 14 days of culture. Bar graphs show the average total erythroid colony numbers, which were calculated by multiplying the frequency times 1.4 to reflect higher viable cell numbers in miR‐513a OE‐HSPCs compared to empty vector control‐HSPCs after 3 days of transduction, from three independent experiments (Mean ± SEM, *n* = 3). (D) On day 3 or day 6 of erythroid culture, average viable cell numbers determined by trypan blue dye exclusion assay are plotted (Mean ± SEM, *n* = 3). Statistical significance was determined by Student's *t*‐test (*P < 0.05). (E) Cell pellets from empty vector‐HSPCs versus miR‐513a OE‐HSPCs cultured in EPO‐containing media for 6 days. (F) On day 6 of erythroid culture, mRNA expression of erythroid genes was assessed by qRT‐PCR and normalized to GAPDH mRNA levels. The average mRNA level of each indicated gene in miR‐513a OE‐HSPCs is plotted relative to control empty vector‐HSPCs set to 1 (Mean ± SEM, *n* = 3). Statistical significance was determined by Student's *t*‐test (*P < 0.05).

To investigate the role of miR‐513a in human erythropoiesis, HSPCs were transduced with control empty vector (EV) or miR‐513a overexpression (OE) lentivirus (LV) (Fig. 2A). The ability of miR‐513a OE LV to express mature miR‐513a‐5p in HSPCs was confirmed by qRT‐PCR (Fig. S4). Transduced HSPCs were cultured in media containing SCF and EPO and assayed for erythroid differentiation after 3 or 6 days of culture. On Day 3 of erythroid culture, miR‐513a OE‐HSPCs had higher percentages of erythroid cells (CD71^hi^CD235a^hi^/CD71^hi^CD105^hi^) as compared to EV‐HSPCs. No differences were observed in the number of cells retaining CD34 expression (Fig. 2B). To evaluate the colony forming efficiency of miR‐513a OE‐HSPCs, transduced HSPCs were plated in methylcellulose media containing SCF and EPO. Total erythroid colony numbers of miR‐513a OE‐HSPCs were slightly higher than EV‐HSPCs, but the difference did not achieve significance (Fig. 2C). Viable cell numbers in miR‐513a OE‐HSPCs were significantly higher than EV‐HSPCs on Day 6 of erythroid culture (Fig. 2D), consistent with previously published reports that proliferation and differentiation of erythroid cells occurs concurrently early in normal erythropoiesis [3, 4]. On Day 6 of culture, enforced miR‐513a expression stimulated hemoglobin synthesis as evidenced by the intense red color cell pellet (Fig. 2E) and increased mRNA expression of all three types of hemoglobin (HBB, HBG and HBA), as compared to EV‐HSPCs (Fig. 2F). In addition, mRNA expression of other erythroid molecules, including ALAS2 and SLC4a1, was significantly higher in miR‐513a OE‐HSPCs than EV‐HSPCs. Although GATA1 mRNA expression was not changed, GATA2 mRNA expression was lower in miR‐513a OE‐HSPCs than EV‐HSPCs (Fig. 2F). These findings indicate that miR‐513a is sufficient to enhance erythropoiesis of human HSPCs.

### MiR‐513a promotes erythroid differentiation in TF‐1 cells, even in the absence of EPO

**Fig. 3:**
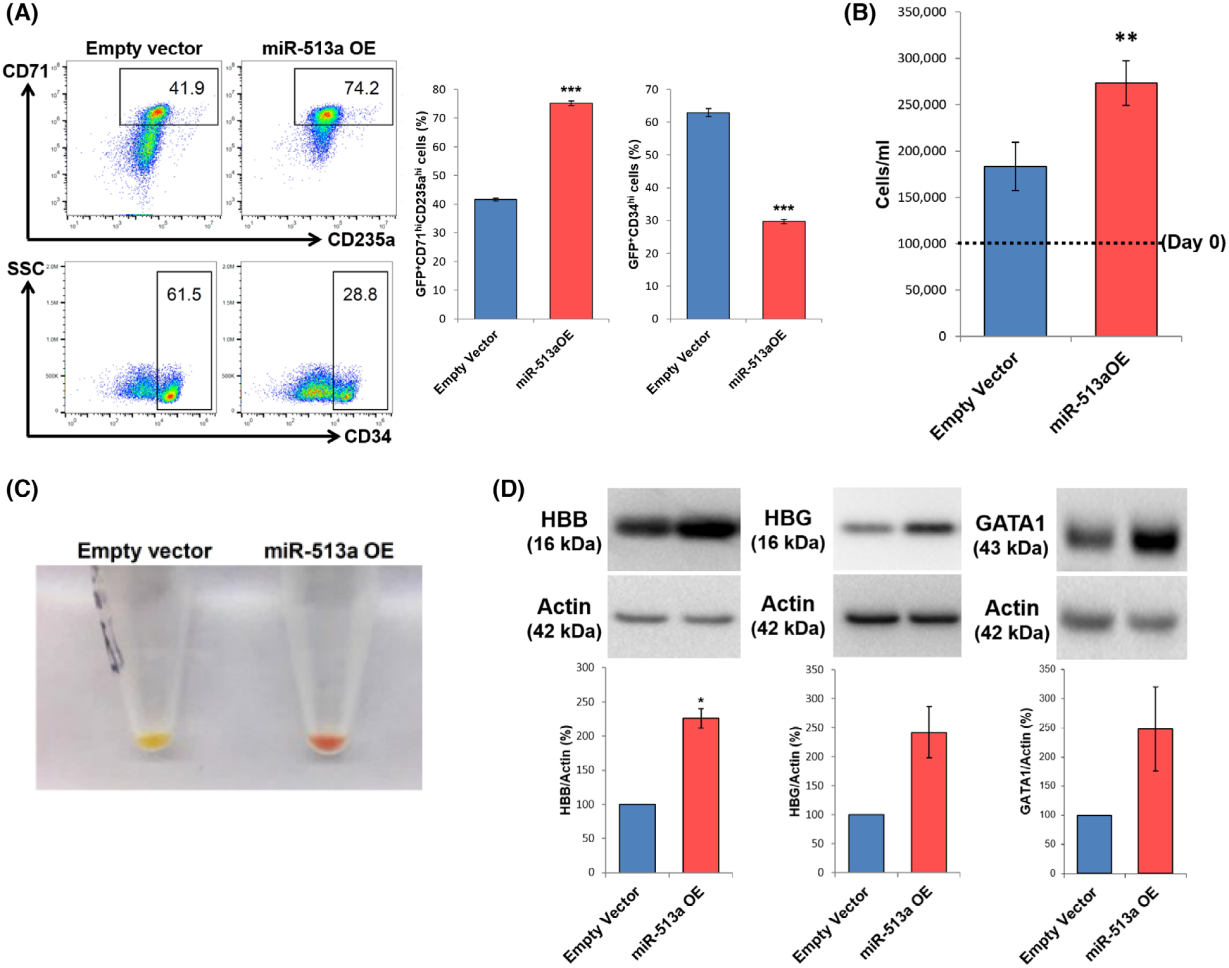
Enforced miR‐513a expression enhances EPO‐stimulated erythroid differentiation in TF‐1 cells. TF‐1 cells transduced with miR empty vector or miR‐513a OE lentivirus were cultured in the EPO‐containing media for 3 days. (A) Transduced TF‐1 cells were immunostained with anti‐CD71, CD235a, and CD34 antibodies. FACS plots are from a representative experiment. The numbers in the boxes indicate %CD71^hi^CD235a^hi^ (top) or %CD34^hi^ (bottom) in the GFP^+^ cell population. Bar graphs show average %CD71^hi^CD235a^hi^ (left) or %CD34^hi^ (right) from three independent experiments (Mean ± SEM, *n* = 3). Statistical significance was determined by Student's *t*‐test (***P < 0.0005). (B) The bar graph shows the average viable cell numbers determined by trypan blue dye exclusion assay (Mean ± SEM, *n* = 3). The dashed line indicates starting cell number on day 0. Statistical significance was determined by Student's *t*‐test (**P < 0.005). (C) Cell pellet photo of miR empty vector‐TF‐1 cells versus miR‐513a OE‐TF‐1 cells cultured in the presence of EPO for 3 days. (D) Transduced TF‐1 cells were analyzed by western blots for HBB, HBG, GATA1, and β‐Actin (endogenous control) protein levels. Western blots are from a representative experiment. Bar graphs show average normalized protein expression levels determined by densitometry from three independent experiments, with empty vector transduced cells set to 100% (Mean ± SEM, *n* = 3). Statistical significance was determined by Student's *t*‐test (*P < 0.05).

Similar to erythropoiesis in HSPCs, EPO stimulates TF‐1 erythroleukemia cells to initiate erythropoiesis, generating cells with increased cell surface expression of the CD71 and CD235a erythroid markers, decreased surface expression of the CD34 HSPC marker, and increased expression of multiple erythroid‐associated RNAs and proteins including hemoglobins (HBB and HBG) [25, 28]. As observed in HSPCs, miR‐513a‐5p expression in TF‐1 cells was increased during early days of EPO‐stimulated erythroid culture (Fig. S5). On Day 3 of erythroid culture, enforced expression of miR‐513a in TF‐1 cells enhanced EPO‐stimulated erythroid differentiation, leading to an increased percentage of erythroid (CD71^hi^CD235a^hi^) cells and higher viable cell numbers (Fig. 3A,B). MiR‐513a OE‐TF‐1 cells also exhibited increased hemoglobin and GATA1 levels, as evidenced by intense red color cell pellets and western blots (Fig. 3C,D). Increased miR‐513a‐5p expression level in miR‐513a OE‐TF‐1 cells was confirmed by qRT‐PCR (Fig. S4). Since we observed similar enhanced erythropoiesis in miR‐513a OE‐TF‐1 cells to those observed in miR‐513a OE‐HSPCs, we therefore utilized TF‐1 cells to further investigate the mechanism by which miR‐513a regulates erythropoiesis.

**Fig. 4:**
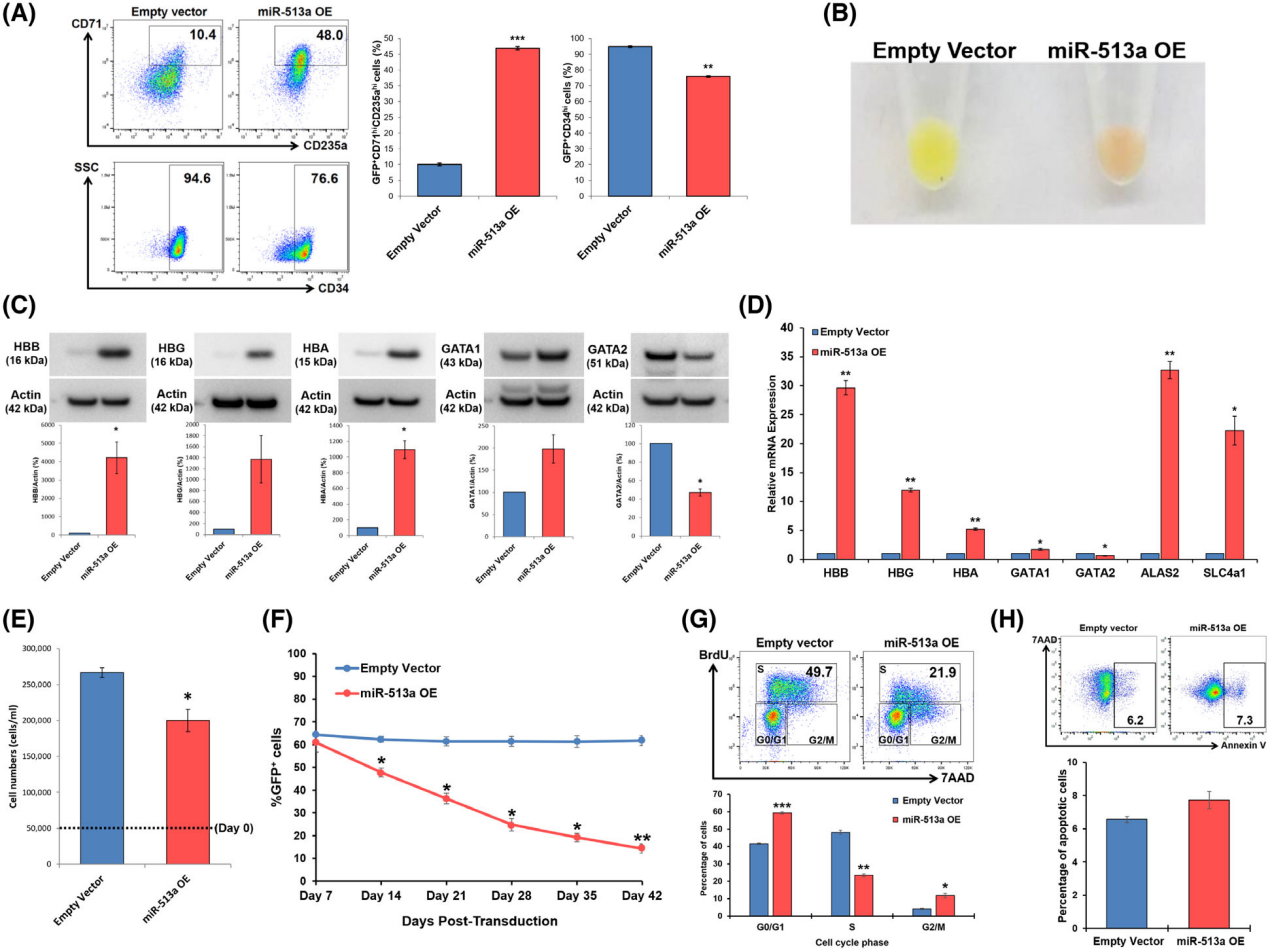
Enforced miR‐513a expression promotes erythroid differentiation in TF‐1 cells in the absence of EPO. (A–D) TF‐1 cells transduced with miR empty vector or miR‐513a OE lentivirus were cultured in the standard GM‐CSF‐containing media (without EPO) for 5 days. (A) Transduced TF‐1 cells were immunostained with anti‐CD71, CD235a, and CD34 antibodies. FACS plots are from a representative experiment. The numbers in the boxes indicate %CD71^hi^CD235a^hi^ (top) or %CD34^hi^ (bottom) in GFP^+^ cells. Bar graphs show average %CD71^hi^CD235a^hi^ (left) or %CD34^hi^ (right) from three independent experiments (Mean ± SEM, *n* = 3). Statistical significance was determined by Student's *t*‐test (**P < 0.005, ***P < 0.0005). (B) Cell pellet of miR empty vector‐TF‐1 cells versus miR‐513a OE‐TF‐1 cells cultured in the standard GM‐CSF media (without EPO). (C) Transduced TF‐1 cells were analyzed by western blot for HBB, HBG, HBA, GATA1, GATA2, and β‐Actin (endogenous control) protein levels. Western blots are from a representative experiment. Bar graphs show normalized protein expression levels determined by densitometry from three independent experiments, with empty vector transduced cells set to 100% (Mean ± SEM, *n* = 3). Statistical significance was determined by Student's *t*‐test (*P < 0.05). (D) Expression of erythroid genes was determined by qRT‐PCR and normalized to GAPDH mRNA levels. Relative mRNA expression of indicated genes in miR‐513a OE‐TF‐1 cells is plotted with the expression levels of empty vector control set to 1 (Mean ± SEM, *n* = 3). Statistical significance was determined by Student's *t*‐test (*P < 0.05, **P < 0.005). (E) At 3 days post‐transduction, transduced TF‐1 cells were plated at 50000 cells/ml and cultured in the standard GM‐CSF media for 2 days. The bar graph shows average viable cell numbers determined by trypan blue dye exclusion assay (Mean ± SEM, *n* = 3). The dashed line indicates starting cell number on day 0. Statistical significance was determined by Student's *t*‐test (*P < 0.05). (F) GFP growth competition assays were performed on TF‐1 cells transduced with miR empty vector versus miR‐513a OE lentivirus (MOI = 0.5). Transduced cells were cultured in the standard GM‐CSF media for 6 weeks and %GFP^+^ cells were assessed weekly by flow cytometry (Mean ± SEM, *n* = 3). Statistical significance was determined by Student's *t*‐test (*P < 0.05, **P < 0.005). (G, H) At 3 days post‐transduction, transduced TF‐1 cells were plated at 50000 cells/ml and cultured in the standard GM‐CSF media for 2 days. (G) Representative FACS plots show cell cycle distribution of transduced TF‐1 cells, with each phase of the cell cycle boxed as indicated (top). The numbers in the boxes indicate the percentage of cells in S phase. Average frequency of cells at each phase of cell cycle from three independent experiments plotted (Mean ± SEM, *n* = 3). Statistical significance was determined by Student's *t*‐test (*P < 0.05, **P < 0.005, ***P < 0.0005). (H) In the representative FACS plots, the percentages of apoptotic cells are boxed, as indicated (top). Average frequency of apoptotic cells from three independent experiments plotted (Mean ± SEM, *n* = 3).

To assess the effect of miR‐513a on erythroid differentiation in the absence of EPO, miR‐513a OE‐TF‐1 cells were cultured in standard TF‐1 culture media (containing 1 ng·mL GM‐CSF) but lacking EPO. On Day 5 of standard culture, miR‐513a OE‐TF‐1 cells generated higher frequencies of erythroid (CD71^hi^CD235a^hi^) cells and lower frequencies of CD34^hi^ cells, as compared to control EV‐TF‐1 cells (Fig. 4A). Even in the absence of EPO, miR‐513a OE‐TF‐1 cells had a red‐colored pellet (Fig. 4B) and increased hemoglobins (HBB, HBG, and HBA) protein levels (Fig. 4C), as compared to EV‐TF‐1 cells. In addition, miR‐513 OE‐TF‐1 cells showed obvious signs of the initiation of erythroid differentiation relative to EV‐TF‐1 cells, with increased GATA1 protein/mRNA expression and reduced GATA2 protein/mRNA expression (Fig. 4C,D). Similarly, mRNA expressions of hemoglobins (HBB, HBG, and HBA), ALAS2, and SLC4a1 were significantly increased in miR‐513a OE‐TF‐1 cells, as compared to EV‐TF‐1 cells (Fig. 4D). Thus, enforced miR‐513a expression in TF‐1 cells stimulates erythroid differentiation, even in the absence of EPO. In addition to TF‐1 cells, we found that enforced miR‐513a expression stimulated erythroid differentiation in K562 leukemia cell line without chemical compounds (e.g., hemin, butyric acid, 5‐azacytidine, or cytosine arabinoside) [31], as evidenced by intense red color cell pellet and increased HBB, HBG, and GATA1 protein levels (Fig. S6A,S6B).

In contrast to miR‐513a OE in HSPCs or TF‐1 cells cultured in the presence of EPO (Figs 2D and 3B), viable cell numbers in miR‐513a OE‐TF‐1 cells or miR‐513a OE‐K562 cells were lower in the standard culture (without EPO), as compared to EV‐TF‐1 cells or EV‐K562 cells (Figs 4E and S6C). To confirm the growth inhibition effect of miR‐513a OE, the GFP competition assay was performed. During 6 weeks of the standard GM‐CSF culture, the %GFP^+^ cells in the miR‐513a OE‐TF‐1 cell population decreased from 60% on Day 7 of post‐transduction to 24.8% on Day 28 and then to 14.3% on Day 42 (Fig. 4F). In contrast, for control EV‐TF‐1 cells, the %GFP^+^ cells remained stable at ~ 60% over the 6 weeks of the GFP competition assay (Fig. 4F). To understand whether the miR‐513a‐mediated growth disadvantage in TF‐1 cells is due to the cell cycle disruption and/or increased apoptosis, we conducted BrdU/7‐AAD staining and AnnexinV/7‐AAD staining. The BrdU/7‐AAD staining analysis showed that miR‐513a OE‐TF‐1 cells had a lower percentage of cells in S‐phase and a higher percentage of cells in G0/G1 and G2/M phases, as compared to EV‐TF‐1 cells (Fig. 4G). Percentages of apoptotic cells in miR‐513a OE‐TF‐1 cells did not differ from EV‐TF‐1 cells (Fig. 4H). These results indicate that miR‐513a OE inhibited TF‐1 cell growth in the absence of EPO, and that this growth disadvantage is due to disrupted cell cycling rather than increased apoptosis.

Since our miR‐513a OE lentivector produces both mature miR‐513a‐5p and miR‐513a‐3p, we next sought to determine which strand mediates the erythroid effect of miR‐513a OE in TF‐1 cells. To further examine this, we constructed two mutant OE lentivectors (Fig. S7A). MiR‐513a‐5p^mut^ contains 2 base mutations in mature miR‐513a‐5p sequences, designed to disrupt miR‐513a‐5p function, whereas similarly designed miR‐513a‐3p^mut^ can disrupt miR‐513a‐3p function. TF‐1 cells were transduced with EV, miR‐513a OE, miR‐513a‐5p^mut^, or miR‐513a‐3p^mut^ LVs, and then transduced TF‐1 cells were cultured in the standard GM‐CSF media (without EPO) for 2 days. TF‐1 cells transduced with miR‐513a‐5p^mut^ LV had lower percentages of erythroid (CD71^hi^CD235a^hi^) cells and higher percentages of CD34^hi^ cells than miR‐513a OE‐TF‐1 cells or miR‐513a‐3p^mut^ TF‐1 cells (Fig. S7B). In addition, miR‐513a‐5p^mut^ OE‐TF‐1 cells had reduced hemoglobins (HBB and HBG) and GATA1 protein levels, as compared to miR‐513a OE‐TF‐1 cells or miR‐513a‐3p^mut^ TF‐1 cells (Fig. S7C). These results support a role for miR‐513a‐5p, rather than miR‐513a‐3p, in promoting erythroid differentiation in TF‐1 cells.

Taken together, our findings indicate that miR‐513a OE can enhance erythroid differentiation even in the absence of EPO and that this effect is largely mediated by miR‐513a‐5p.

### MiR‐513a‐mediated erythroid differentiation in TF‐1 cells requires GATA1 but not EPOR

Since the enforced miR‐513a expression in TF‐1 cells stimulates erythroid differentiation and increases GATA1 expression even in the absence of EPO, we tested whether EPOR or GATA1 is required for miR‐513a‐mediated TF‐1 erythropoiesis.

**Fig. 5:**
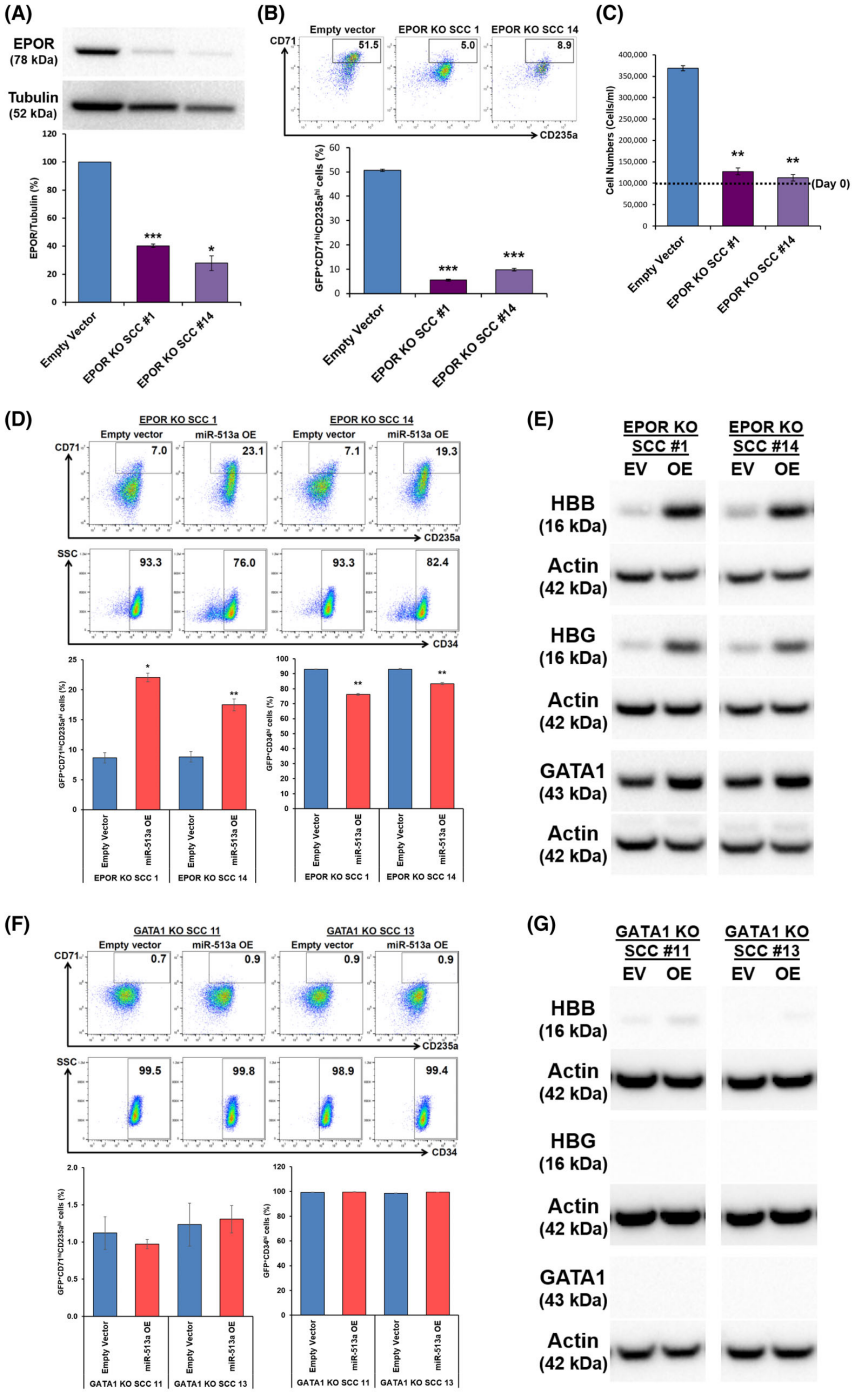
MiR‐513a‐mediated erythroid differentiation in TF‐1 cells requires GATA1 but not EPOR. (A‐C) CRISPR/Cas9‐mediated EPOR KO TF‐1 cells were cultured in the EPO‐containing media for 2 days. (A) EPOR KO TF‐1 cells were analyzed by western blots for EPOR and α‐tubulin (endogenous control) protein levels. The western blot image is from a representative experiment. The bar graph shows average normalized protein expression levels determined by densitometry from three independent experiments (Mean ± SEM, *n* = 3). Statistical significance was determined by Student's *t*‐test (*P < 0.05, ***P < 0.0005). (B) EPOR KO TF‐1 cells were immunostained with anti‐CD71 and CD235a antibodies. FACS plots are from a representative experiment. The bar graph shows average %CD71^hi^CD235a^hi^ in GFP^+^ cells from three independent experiments (Mean ± SEM, *n* = 3). Statistical significance was determined by Student's *t*‐test (***P < 0.0005). (C) Average viable cell numbers determined by trypan blue dye exclusion assay plotted (Mean ± SEM, *n* = 3). The dashed line indicates starting cell number on day 0. Statistical significance was determined by Student's *t*‐test (**P < 0.005**). (D‐G) EPOR KO TF‐1 SCCs (2 clones; #1 and #14) or GATA1 KO TF‐1 SCCs (2 clones; #11 and #13) were transduced with miR empty vector or miR‐513a OE lentivirus prior to culturing in the standard GM‐CSF media (without EPO) for 8 days. (D, F) Transduced TF‐1 cells were immunostained with anti‐CD71, CD235a, and CD34 antibodies. FACS plots are from a representative experiment. The numbers in the boxes indicate %CD71^hi^CD235a^hi^ (top) or %CD34^hi^ (bottom) in the GFP^+^ cell population. Bar graphs show average %CD71^hi^CD235a^hi^ (left) or average %CD34^hi^ (right) from three independent experiments (Mean ± SEM, *n* = 3). Statistical significance was determined by Student's *t*‐test (*P < 0.05, **P < 0.005). (E, G) Transduced TF‐1 cells were analyzed by western blots for HBB, HBG, GATA1, and β‐Actin (endogenous control) protein levels. Western blots from a representative experiment are shown (*n* = 3).

First, two independent EPOR knockout (KO) single cell clones (EPOR KO SCC #1 and EPOR KO SCC #14) were generated from TF‐1 cells using CRISPR/Cas9 KO technology. KO efficiency was confirmed by genomic DNA sequencing, followed by Synthego Inference of CRISPR Edits (ICE) analysis [32]. Based on the ICE analysis, the KO score of EPOR KO SCC #1 and EPOR KO SCC #14 is 56% and 82%, respectively. Western blots confirmed that EPOR protein levels decreased by 60% in EPOR KO SCC #1 and 73% in EPOR KO SCC #14 (Fig. 5A). In addition, the percentage of erythroid (CD71^hi^CD235a^hi^) cells and the viable cell numbers in EPOR KO TF‐1 cells did not increase during EPO‐stimulated erythroid cultures (Fig. 5B,C), indicating that TF‐1 cells cannot differentiate into erythroid cells in the absence of EPOR. These EPOR KO TF‐1 cells were transduced with EV LV or miR‐513a OE LV, and then the resulting miR‐513aOE/EPOR KO TF‐1 cells were cultured in standard GM‐CSF media (without EPO) for 8 days. Enforced miR‐513a expression in EPOR KO TF‐1 cells resulted in higher percentages of erythroid cells and lower percentages of CD34^hi^ cells than control cells (Fig. 5D). In addition, we observed higher HBB, HBG, and GATA1 protein levels in miR‐513aOE/EPOR KO TF‐1 cells, compared to controls (Figs 5E and S8A). Thus, miR‐513a‐stimulated erythroid differentiation is independent of EPOR, supporting our finding that miR‐513a enhances erythroid differentiation in the absence of EPO.

Next, two independent GATA1 KO TF‐1 single cell clones (GATA1 KO SCC #11 and GATA1 KO SCC #13), which had been used in our previous study [25], were transduced with EV LV or miR‐513a OE LV, and then miR‐513a OE/GATA1 KO TF‐1 cells were cultured in the standard GM‐CSF media (without EPO) for 8 days. No significant differences were observed in the surface marker expression of CD71, CD235a and CD34, or protein expressions of hemoglobin and GATA1 in miR‐513a OE/GATA1 KO TF‐1 cells, as compared to control cells (Fig. 5F,G and Fig. S8B). Thus, miR‐513a OE was not able to stimulate erythropoiesis in GATA1‐deficient TF‐1 cells, indicating a requirement for GATA1.

### MiR‐513a reduces the expression of c‐Jun and phospho‐c‐Jun to promote human erythropoiesis

To identify potential targets of miR‐513a‐5p, we combined the predicted target list from the RNA22v2.0 target prediction tool [33] with our mRNA microarray data (Fig. S9A and Table S5). Of the 53 predicted miR‐513a‐5p targets from the intersection of these datasets, we focused on c‐JUN, which had been previously reported as a negative regulator of erythropoiesis by GATA1 repression [34].

**Fig. 6:**
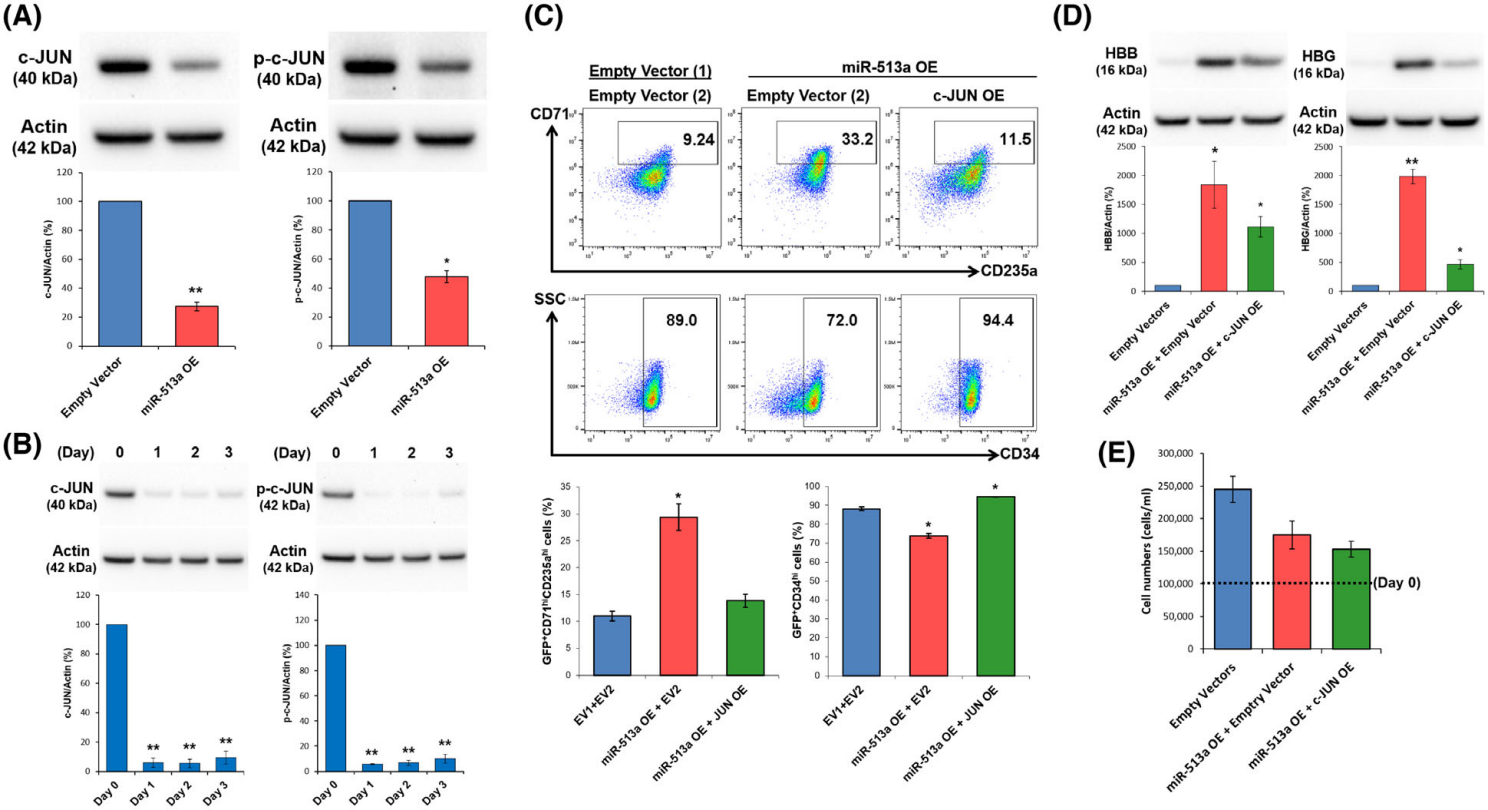
MiR‐513a‐mediated reduction of c‐Jun and phospho‐c‐Jun enhances erythroid differentiation in TF‐1 cells. (A) TF‐1 cells transduced with miR empty vector or miR‐513a OE lentivirus were cultured in the standard GM‐CSF media (in the absence of EPO). On day 5 of standard culture, transduced cells were analyzed by western blots for c‐Jun, phospho‐c‐Jun, and β‐Actin (endogenous control) protein levels. Western blots are from a representative experiment. Bar graphs show average normalized protein expression levels determined by densitometry from three independent experiments (Mean ± SEM, *n* = 3). Statistical significance was determined by Student's *t*‐test (*P < 0.05, **P < 0.005). (B) Whole cell protein lysates were prepared from TF‐1 cells cultured in EPO‐containing media on day 0, 1, 2, and 3. Protein expression levels of c‐Jun, phospho‐c‐Jun, and β‐Actin (endogenous control) were determined by western blot analysis. Western blots are from a representative experiment. Bar graphs show average normalized protein expression levels determined by densitometry from three independent experiments (Mean ± SEM, *n* = 3). Statistical significance was determined by Student's *t*‐test (**P < 0.005**). (C–E) TF‐1 cells double‐transduced with miR‐513a OE lentivirus and c‐JUN OE lentivirus were cultured in standard GM‐CSF media for 8 days. (C) Transduced TF‐1 cells were immunostained with anti‐CD71, CD235a, and CD34 antibodies. FACS plots are from a representative experiment. The numbers in the boxes indicate %CD71^hi^CD235a^hi^ (top) or %CD34^hi^ (bottom) in GFP^+^ cells. Bar graphs show average %CD71^hi^CD235a^hi^ (left) or %CD34^hi^ (right) from three independent experiments (Mean ± SEM, *n* = 3). Statistical significance was determined by Student's *t*‐test (*P < 0.05). (D) Transduced TF‐1 cells were analyzed by western blots for HBB, HBG, and β‐Actin (endogenous control) protein levels. Western blots are from a representative experiment. Bar graphs show average normalized protein expression levels determined by densitometry from three independent experiments (Mean ± SEM, *n* = 3). Statistical significance was determined by Student's *t*‐test (*P < 0.05, **P < 0.005). (E) Average viable cell numbers determined by trypan blue dye exclusion assay plotted (Mean ± SEM, *n* = 3). The dashed line indicates starting cell number on day 0.

First, we confirmed that miR‐513a OE‐TF‐1 cells and miR‐513a OE‐K562 cells had lower endogenous c‐Jun and lower phosphorylated c‐Jun (phospho‐c‐Jun) protein levels (Figs 6A and S6A). In addition, endogenous protein levels of c‐Jun and phospho‐c‐Jun decreased during the early days of EPO‐stimulated TF‐1 erythropoiesis (Fig. 6B).

To examine whether c‐JUN is a direct target of miR‐513a‐5p, we performed luciferase assays. A partial human c‐JUN 3′UTR, containing one predicted miR‐513a‐5p binding site, was cloned downstream of the Firefly luciferase gene (subsequently referred to as WT). A mutant construct was generated by deleting the seed sequences (10‐bp deletion) from the predicted miR‐513a‐5p binding site (Fig. S9B). Relative luciferase activity of c‐JUN 3′UTR constructs was assessed following cotransfection of miR‐513a‐5p mimics in HEK293T cells. We did not observe differences in relative luciferase activity of c‐JUN 3′UTR constructs (WT versus mutant) after co‐transfection of miR‐513a‐5p mimics (Fig. S9C), providing evidence that c‐JUN is not a direct target of miR‐513a‐5p.

Next, we performed a ‘rescue’ experiment to determine whether c‐JUN functions downstream of miR‐513a to mediate enhanced erythropoiesis. TF‐1 cells were co‐transduced with miR‐513a OE LV and c‐JUN OE LV (Fig. S9D), and restored c‐Jun protein expression in c‐JUN OE/miR‐513a OE‐TF‐1 cells was confirmed by western blots (Fig. S9E). After 8 days of the standard GM‐CSF culture, double‐transduced c‐JUN OE/miR‐513a OE‐TF‐1 cells generated lower percentages of erythroid (CD71^hi^CD235a^hi^) cells and higher percentages of CD34^hi^ cells, as compared to cells double‐transduced with miR‐513a OE LV and EV LV (Fig. 6C). In addition, we observed lower HBB and HBG protein levels in c‐JUN OE/miR‐513a OE‐TF‐1 cells, as compared to control miR‐513a OE/EV‐TF‐1 cells (Fig. 6D). These results suggest that c‐JUN OE partially reversed miR‐513a‐mediated enhancement of TF‐1 erythropoiesis. Notably, the decreased viable cell numbers in miR‐513a OE‐TF‐1 cells were not restored by c‐JUN OE (Fig. 6E).

**Fig. 7:**
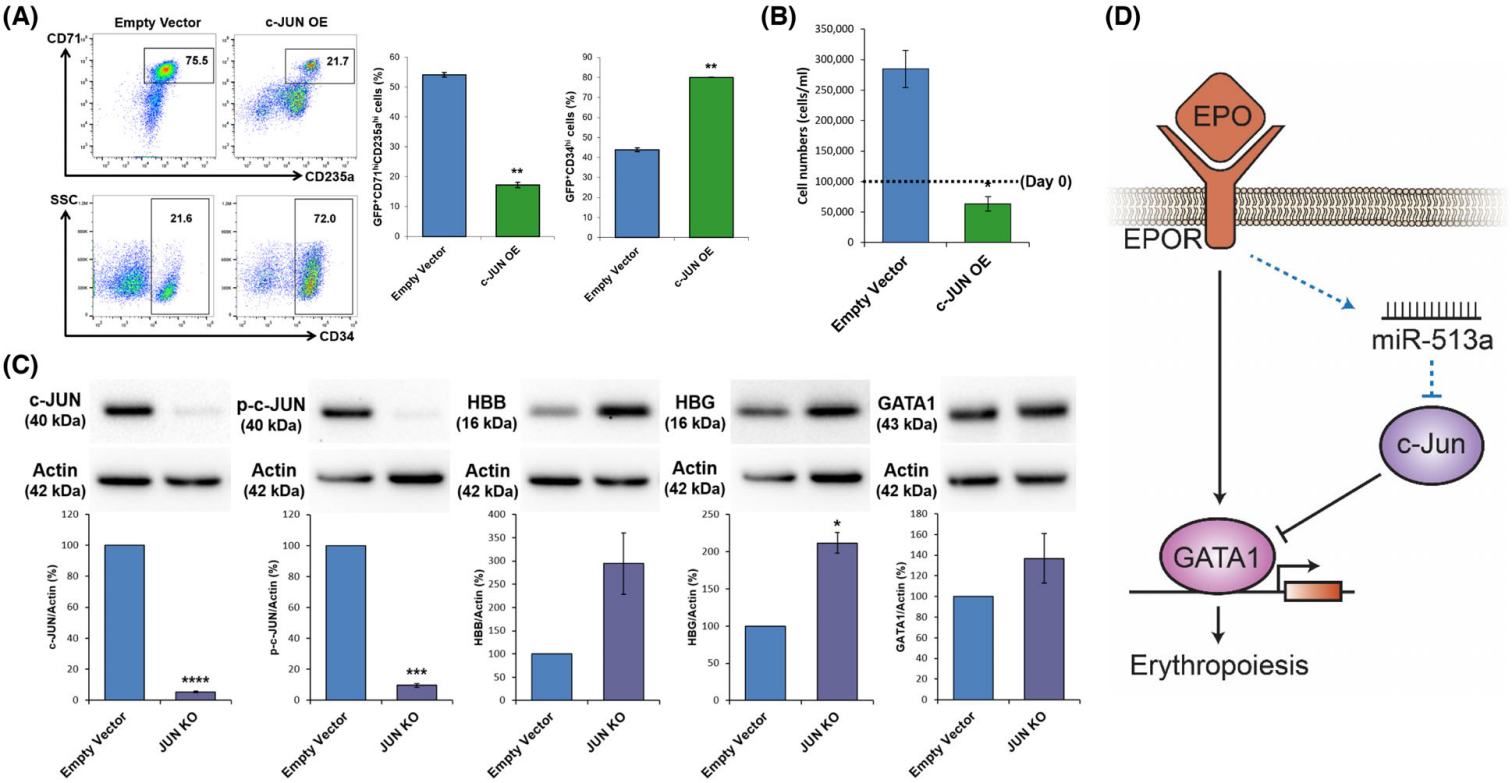
c‐JUN regulates erythroid differentiation in TF‐1 cells. (A, B) TF‐1 cells transduced with empty vector or c‐JUN OE lentivirus were cultured in EPO‐containing media for 5 days. Transduced TF‐1 cells were immunostained with anti‐CD71, CD235a, and CD34 antibodies and FACS plots are from a representative experiment. The numbers in the boxes indicate %CD71^hi^CD235a^hi^ (top) or %CD34^hi^ (bottom) in GFP^+^ cells. Bar graphs show average %CD71^hi^CD235a^hi^ (left) or %CD34^hi^ (right) from three independent experiments (Mean ± SEM, *n* = 3). Statistical significance was determined by Student's *t*‐test (**P < 0.005). (B) Average viable cell numbers determined by trypan blue dye exclusion assay plotted (Mean ± SEM, *n* = 3). The dashed line indicates starting cell number on day 0. Statistical significance was determined by Student's *t*‐test (*P < 0.05). (C) CRISPR/Cas9‐mediated c‐JUN KO TF‐1 cells were cultured in the standard GM‐CSF media for 5 days. Protein expression levels of c‐Jun, phospho‐c‐Jun, HBB, HBG, GATA1, and β‐Actin (endogenous control) were determined by western blots. Western blots are from a representative experiment. Bar graphs show average normalized protein levels determined by densitometry from three independent experiments (Mean ± SEM, *n* = 3). Statistical significance was determined by Student's *t*‐test (*P < 0.05, ***P < 0.0005, ****P < 0.00005). (D) Proposed model for miR‐513a as a positive regulator of erythroid differentiation. During early human erythropoiesis, miR‐513a promotes erythroid differentiation, at least in part, by indirectly decreasing c‐Jun expression, which is associated with increased GATA1 expression. Black solid lines indicate previously reported interactions, and blue dashed lines indicate interactions demonstrated in this study.

Consistent with published reports [34, 35, 36], c‐JUN OE reduced EPO‐stimulated TF‐1 erythropoiesis, as indicated by lower percentages of erythroid (CD71^hi^CD235a^hi^) cells and higher percentages of CD34^hi^ cells than in control cells (Fig. 7A). Viable cell numbers in c‐JUN OE‐TF‐1 cells were significantly lower than EV‐TF‐1 cells on day 5 of EPO‐stimulated erythroid culture (Fig. 7B). To further examine the role of c‐JUN, c‐JUN KO TF‐1 cells were generated using LV‐mediated CRISPR/Cas9 KO technology. The KO efficiency was confirmed by the ICE analysis [32], where the average KO score of c‐JUN KO TF‐1 bulk population was 77 ± 1% (average ± SEM, p = 0.00006, *n* = 3). Western blots confirmed that both c‐Jun and phospho‐c‐Jun protein levels decreased by 95% and 91%, respectively, in the c‐JUN KO TF‐1 bulk population (Fig. 7C). In addition, c‐JUN KO TF‐1 cells had higher HBB, HBG, and GATA1 protein levels when cultured in the absence of EPO, as compared to controls (Fig. 7C). However, the percentage of erythroid (CD34^low^CD71^hi^CD235a^hi^) cells and viable cell numbers in c‐JUN KO TF‐1 cells did not differ from controls, regardless of the presence or absence of EPO (Figs S9F–S9I).

Together, these results indicate that miR‐513a‐mediated reduction of c‐Jun and phospho‐c‐Jun likely contributes to enhanced hemoglobin expression in erythroid cells, while having minimal effect on surface erythroid marker expression or erythroid cell growth.

## Discussion

MiRs regulate numerous cellular and developmental processes including erythropoiesis, and distinct miR expression patterns are associated with specific stages of differentiation. Although several groups have conducted miR expression profiling studies of erythroid cells and reported multiple miRs that regulate normal erythropoiesis [11, 16], most of these studies focused on miRs that are differentially expressed between undifferentiated cells and very late erythroid cells, for example, primary HSPCs cultured in erythroid media for 9–15 days [19, 20, 21, 22, 23, 24]. In this study, we sought to identify miRs that might regulate early stages of normal erythropoiesis by comparing miR expression between a population of FACS‐sorted early erythroid progenitor/precursor cells (EE), which is enriched in erythroid colony‐forming cells, and uncultured primary HSPCs (E0). Our miR profiling analysis identified 40 differentially expressed miRs, including 12 upregulated and 28 downregulated miRs in the early erythroid (EE) population compared with the uncultured HSPC (E0) population. Consistent with prior reports [11, 16, 17, 18], the expression of miR‐144, miR‐451 and miR‐486 was upregulated in the EE population (Fig. S3A). Among 12 upregulated miRs, we focused on miR‐513a‐5p, as it had not previously been studied in the context of erythropoiesis. It has been reported that miR‐513a‐5p functions as a tumor suppressor in osteosarcoma cells, retinoblastoma cells, or renal cell carcinoma [37, 38, 39, 40], whereas it acts in oncogenic manner in glioma, nasopharyngeal cancer, or breast cancer cells [41, 42, 43, 44]. MiR‐513a‐5p is also reported as a biomarker in high‐risk breast cancer [43].

In this study, we found that expression of miR‐513a‐5p increased early during erythroid differentiation in both HSPCs and TF‐1 cells (Figs 1C and S5). In the presence of EPO, enforced miR‐513a expression resulted in increased numbers of erythroid cells, as well as increased levels of several established erythroid marker proteins and mRNAs, including hemoglobins and GATA1 in HSPCs and TF‐1 cells (Figs 2 and 3). Interestingly, enforced miR‐513a expression in TF‐1 cells also promoted the generation of erythroid (CD34^low^CD71^hi^CD235a^hi^) cells in standard GM‐CSF culture (which lacks EPO) (Fig. 4A). Along with increased erythroid surface marker expressions, hemoglobin protein and mRNA levels were increased in miR‐513a OE‐TF‐1 cells, even in the absence of EPO (Fig. 4B,D). Furthermore, disruption of seed sequences in mature miR‐513a‐5p abolished this enhanced erythroid differentiation effect in TF‐1 cells (Fig. S7). Together, these findings support the role of miR‐513a‐5p in promoting erythroid differentiation in TF‐1 cells, even in the absence of EPO.

It has been reported that erythroid differentiation and proliferation occur simultaneously at the early stage of erythropoiesis [3, 4, 5, 6]. MiR‐513a OE in both HSPCs and TF‐1 cells increased viable cell numbers during erythroid culture in the presence of EPO (Figs 2D and 3B). In contrast, miR‐513a OE in TF‐1 cells cultured in the standard GM‐CSF media (without EPO) decreased viable cell numbers due to disrupted cell cycling (Figs 4E–H), suggesting that the miR‐513a‐associated effect on erythroid cell proliferation is dependent on the presence of EPO. Recently, the CDK‐inhibitor p27^kip1^, which regulates CDK kinase activities during the G1‐S transition [45], has been reported to play a crucial role in erythroid progenitor proliferation during early erythropoiesis [46]. In p27^Y88F/Y88F^ knock‐in mice, impaired phosphorylation of p27 led to decreased proliferation of early erythroid progenitors, which may be compensated by increased EPO levels [46]. This p27‐associated effect on early erythroid cell proliferation is similar to our observation that miR‐513a OE‐TF‐1 cells showed increased proliferation only in the presence of EPO. Therefore, in the future, it would be interesting to investigate whether miR‐513a regulates erythroid cell proliferation through p27 and/or other CDK regulators.

GATA1, a master transcription factor for erythropoiesis, regulates the expression of many genes essential for erythroid differentiation and proliferation [7]. GATA1 also regulates the expression of miRs, including miR‐144, miR‐451, miR‐27a, miR‐24, and miR‐23a, which play important roles in erythropoiesis [11, 12, 13]. Among these, miR‐24 and miR‐27a have been reported to directly target GATA2 [47], whose expression decreases during erythropoiesis. Therefore, miRs are crucial regulators for the ‘GATA switch’, which is a critical factor in the initiation of erythropoiesis [7, 8, 9, 10]. In this study, miR‐513a OE‐TF‐1 cells showed increased GATA1 expression and decreased GATA2 expression (Fig. 4C,D). We also found that enforced miR‐513a expression increased protein levels of hemoglobins and GATA1 in K562 cells (Fig. S6A). Given that miR‐513a OE‐mediated erythropoiesis is involved in regulating GATA1/GATA2 expression, a key consequence of EPO‐stimulated erythropoiesis, we investigated whether EPOR and/or GATA1 is required for miR‐513a‐mediated erythropoiesis using EPOR KO TF‐1 cells and GATA1 KO TF‐1 cells. We found that miR‐513a OE in EPOR KO TF‐1 cells increased erythroid differentiation, while miR‐513a OE in GATA1 KO TF‐1 cells did not (Figs 5D–G). These results indicate that miR‐513a promotes erythropoiesis in a GATA1‐dependent and EPOR‐independent manner, likely acting upstream of GATA1 and downstream of EPOR. Therefore, miR‐513a may contribute to the “GATA switch” indirectly by modulating the expression of GATA1 and GATA2.

To identify the potential targets of miR‐513a‐5p, we combined bioinformatics analysis of the predicted miR‐513a‐5p target list with our list of differentially expressed erythroid mRNAs (Table S5). Among 53 predicted targets from our bioinformatics analysis (Fig. S9A), we focused on c‐JUN, which has been shown to negatively regulate erythropoiesis [34, 35, 36]. It has been reported that KD of c‐JUN using antisense oligonucleotides induces erythroid differentiation, while c‐JUN OE inhibits DMSO‐induced erythroid differentiation in Friend Murine Erythroleukemia (F‐MEL) cells [35]. Enforced dominant negative c‐JUN expression increases erythroid differentiation of K562 cells [36]. In addition, retrovirus‐mediated c‐JUN OE inhibits erythroid differentiation of primary human HSPCs by increasing the expression of HERP2 (HEY1), which physically interacts with GATA1 to repress its transcriptional activities [34]. Consistent with these reports, we found that c‐JUN OE in TF‐1 cells inhibited EPO‐stimulated erythroid differentiation (Fig. 7A) and CRISPR/Cas9‐mediated c‐JUN KO increased hemoglobin protein expression in TF‐1 cells (Fig. 7C). Furthermore, while c‐JUN was not a direct target of miR‐513a‐5p (Fig. S9B,C), c‐JUN OE abolished miR‐513a‐mediated enhancement of erythropoiesis (Fig. 6C,D). Thus, our data suggest that miR‐513a promotes erythroid differentiation, at least in part, by indirectly decreasing c‐Jun and phospho‐c‐Jun expression.

## Conclusion

In summary, our findings demonstrate a role for miR‐513a as a positive regulator of early human erythropoiesis, associated with reduced c‐Jun and phospho‐c‐Jun expression and increased GATA1 expression (Fig. 7D), probably via multiple targets. To the best of our knowledge, this study is the first to identify miR‐513a as an erythroid‐associated miR that promotes human erythroid differentiation in a GATA1‐dependent manner and enhances erythroid cell proliferation in an EPO‐dependent manner.

## Conflicts of interest

The authors declare no conflicts of interest.

## Author contributions

MK and TJK conceived the study and designed the experiments. MK, BT, SB, CLE, MA, TMC, AY, TLH, TD, SG, and TJK performed research and analyzed data. MK, CLE, SG, CIC, and TJK wrote and revised the manuscript. MK, CIC, and TJK acquired funding for research. All authors read and approved the manuscript submission.

## Supporting information


Appendix S1.
**Table S1.** Primer sequences used for cloning.
**Table S2.** Antibodies used for the erythroid surface marker FACS staining.
**Table S3.** Primer sequences used for qRT‐PCR.
**Table S4.** Antibodies used for the western blot analysis.
**Table S5.** The list of miRs and mRNAs from microarray profiling.
**Figure S1.** FACS gating strategy for assessing erythroid differentiation in HSPCs.
**Figure S2.** MiR‐513a‐5p expression increases during early EPO‐stimulated erythroid differentiation.
**Figure S3.** Differentially expressed miRs in early erythroid cells were identified by microarray profiling.
**Figure S4.** Increased miR‐513a‐5p expression in miR‐513a OE‐HSPCs and miR‐513a OE‐TF‐1 cells confirmed by qRT‐PCR.
**Figure S5.** MiR‐513a‐5p increases early during EPO‐stimulated erythroid differentiation in TF‐1 cells.
**Figure S6.** Enforced miR‐513a expression promotes erythroid differentiation in K562 cells.
**Figure S7.** MiR‐513a‐5p, rather than miR‐513‐3p, promotes erythroid differentiation in TF‐1 cells, in the absence of EPO.
**Figure S8.** MiR‐513a‐mediated erythroid differentiation in TF‐1 cells requires GATA1 but not EPOR.
**Figure S9.** c‐JUN is not a direct target of miR‐513a‐5p, but contributes to miR‐513a‐mediated erythropoiesis.

## Data Availability

Data will be available upon request to the corresponding author.
