# Supplementary material for: MiR‐513a promotes human erythroid differentiation by modulating c‐Jun
Source: FEBS Open Bio. 2026 Mar 8;16(8):1509–26. doi: 10.1002/2211-5463.70226 (PMC13399029; doi:10.1002/2211-5463.70226)
Supplement: Supplementary file 1 — Appendix S1. Table S1. Primer sequences used for cloning. Table S2. Antibodies used for the erythroid surface marker FACS staining. Table S3. Primer sequences used for qRT‐PCR. Table S4. Antibodies used for the western blot analysis. Table S5. The list of miRs and mRNAs from microarray profiling. Figure S1. FACS gating strategy for assessing erythroid differentiation in HSPCs. Figure S2. MiR‐513a‐5p expression increases during early EPO‐stimulated erythroid differentiation. Figure S3. Differentially expressed miRs in early erythroid cells were identified by microarray profiling. Figure S4. Increased miR‐513a‐5p expression in miR‐513a OE‐HSPCs and miR‐513a OE‐TF‐1 cells confirmed by qRT‐PCR. Figure S5. MiR‐513a‐5p increases early during EPO‐stimulated erythroid differentiation in TF‐1 cells. Figure S6. Enforced miR‐513a expression promotes erythroid differentiation in K562 cells. Figure S7. MiR‐513a‐5p, rather than miR‐513‐3p, promotes erythroid differentiation in TF‐1 cells, in the absence of EPO. Figure S8. MiR‐513a‐mediated erythroid differentiation in TF‐1 cells requires GATA1 but not EPOR. Figure S9. c‐JUN is not a direct target of miR‐513a‐5p, but contributes to miR‐513a‐mediated erythropoiesis. [file FEB4-16-1509-s001.zip › feb470226-sup-0001-supinfo.docx]

**Supplementary Information**

**MiR-513a promotes human erythroid differentiation by modulating c-Jun**

MinJung Kim^1,2^, Brittany Taylor^1,3^, Shannon Bolten^1,3^, Christian L. Eberly^1,2^, Mahliya Abdurahman^1,2^, T. Michael Creed^1,2,3^, Acong Yang^4^, Taylor L. Hatchet^1,2^, Tristan Dyson^1,3^, Shuo Gu^4^, Curt I. Civin^1,2,3^, and Tami J. Kingsbury^1,3^*

^1^Center for Stem Cell Biology & Regenerative Medicine, University of Maryland School of Medicine, Baltimore, MD, United States

^2^Department of Pediatrics, University of Maryland School of Medicine, Baltimore, MD, United States

^3^Department of Physiology, University of Maryland School of Medicine, Baltimore, MD, United States

^4^RNA Biology Laboratory, Center for Cancer Research, National Cancer Institute, Frederick, MD, United States

****Disclaimer:*** *This article was prepared while T Kingsbury was employed at University of Maryland School of Medicine. The opinions expressed in this article are the author's own and do not reflect the view of the National Institutes of Health, the Department of Health and Human Services, or the United States government.*

Supplementary Table S1. Primer sequences used for cloning

| **Name** | **Sequences (5' → 3')** | **Enzyme sites** |
| --- | --- | --- |
| miR-513a OE-F | GATATCAGGATGAAAAGGGGAGTG | EcoRV |
| miR-513a OE-R | GGTACCGTTTTCTCAGGAGTCCACA | KpnI |
| miR-513a-5p^mut^ OE-F | CATTCAGCGTACAGTGCCTTTTATAGGGAGGTGTCATTTATGTGA | N/A |
| miR-513a-5p^mut^ OE-R | TCACATAAATGACACCTCCCTATAAAAGGCACTGTACGCTGAATG | N/A |
| miR-513a-3p^mut^ OE-F | GTGTCATTTATGTGAACTAAAATATGAGTTTCACCTTTCTGAGAAGGGT | N/A |
| miR-513a-3p^mut^ OE-R | ACCCTTCTCAGAAAGGTGAAACTCATATTTTAGTTCACATAAATGACAC | N/A |
| c-JUN OE-F | CTGA GCTAGC ATGACTGCAAAGATGGAAACG | NheI |
| c-JUN OE-R | CTGA GAATTC TCAAAATGTTTGCAACTGCTGC | EcoRI |
| c-JunKO-sg1-TOP | CaccgGTAGCCATAAGGTCCGCTCT | BsmBI |
| c-JunKO-sg1-BOT | AaacAGAGCGGACCTTATGGCTACc | BsmBI |
| EPOR KO-sgRNA-TOP | Caccg CGCCTAACCTCCCGGACCC | BsmBI |
| EPOR KO-sgRNA-BOT | Aaac GGGTCCGGGAGGTTAGGCG c | BsmBI |

Supplementary Table S2. Antibodies used for the erythroid surface marker FACS staining

| **Antibody** | **Company** | **Catalog #** |
| --- | --- | --- |
| CD34-APC | BD Biosciences | 555824 |
| CD34-BV650 | BioLegend | 343624 |
| CD71-PECy7 | Thermo Fisher Scientific | 25-0719-42 |
| CD235a-APC | BD Biosciences | 551336 |
| CD105-BV421 | BioLegend | 800510 |

Supplementary Table S3. Primer sequences used for qRT-PCR

| **Gene Name** | **Primer** | **Sequences (5' → 3')** |
| --- | --- | --- |
| Mature miR-513a-5p | miR-513a-5p_F | TTCACAGGGAGGTGTCATTTAT |
| U6 snRNA qPCR Forward (Clontech) | U6 Forward Primer | GGAACGATACAGAGAAGATTAGC |
| U6 snRNA qPCR Reverse (Clontech) | U6 Reverse Primer | TGGAACGCTTCACGAATTTGCG |
| HBB (Beta Hemoglobin) | HBB_F | TCCACTCCTGATGCTGTTATG |
|  | HBB_R | GAGCCAGGCCATCACTAAA |
| HBG (Gamma Hemoglobin) | HBG_F | CTTCTGGAACGTCTGAGGTTATC |
|  | HBG_R | CTCCAGCATCTTCCACATTCA |
| HBA (Alpha Hemoglobin) | HBA_F | CGGTCAACTTCAAGCTCCTAA |
|  | HBA_R | ACAGAAGCCAGGAACTTGTC |
| GATA1 (GATA-Binding Protein 1) | GATA1_F | ACACTGTGGCGGAGAAATG |
|  | GATA1_R | GTTCACCGGGTGTAGCTTGTAG |
| GATA2 (GATA-Binding Protein 2) | GATA2_F | AGTCTGGATCCCTTCCTTCT |
|  | GATA2_R | GACGACAACCACCACCTTAT |
| ALAS2 (Delta-Aminolevulinate Synthase 2) | ALAS2_F | CAACATCTCAGGCACCAGTAAG |
|  | ALAS2_R | CAACAAAGCAGGAGGAGAAGAG |
| SLC4a1 (Solute Carrier Family 4 – Anion Exchanger – Member 1) | SLC4a1_F | CTGTTCAAGCCACCCAAGTA |
|  | SLC4a1_R | GATCTGGATGCCCGTGAATAA |
| GAPDH (Glyceraldehyde-3-Phosphate Dehydrogenase) | GAPDH_F | GGTGTGAACCATGAGAAGTATGA |
|  | GAPDH_R | GAGTCCTTCCACGATACCAAAG |
| β-Actin (Beta-Actin) | Actin_F | TGGACATCCGCAAAGACCTG |
|  | Actin_R | GCCGATCCACACGGAGTACTT |

Supplementary Table S4. Antibodies used for the western blot analysis

| **Antibody** | **Company** | **Catalog #** |
| --- | --- | --- |
| HBB | SantaCruz Biotech | sc-21757 |
| HBG | SantaCruz Biotech | sc-21756 |
| HBA | SantaCruz Biotech | sc-31110 |
| GATA1 | Cell Signaling Technology | 3535S |
| GATA2 | Cell Signaling Technology | 4595S |
| c-JUN | Cell Signaling Technology | 9165S |
| p-c-JUN | Cell Signaling Technology | 9261S |
| EPO Receptor (EPOR) | SantaCruz Biotech | sc-365662 |
| β-Actin (mouse) | Cell Signaling Technology | 3700S |
| β-Actin (rabbit) | Cell Signaling Technology | 8457S |
| α-Tubulin | Cell Signaling Technology | 3873S |
| Horseradish peroxidase (HRP)-conjugated Goat Anti-Mouse IgG | Jackson ImmunoResearch | 115-035-174 |
| HRP-conjugated Goat Anti-Rabbit IgG | Jackson ImmunoResearch | 111-035-003 |
| HRP-conjugated Donkey anti-Rat IgG | Jackson ImmunoResearch | 712-035-153 |


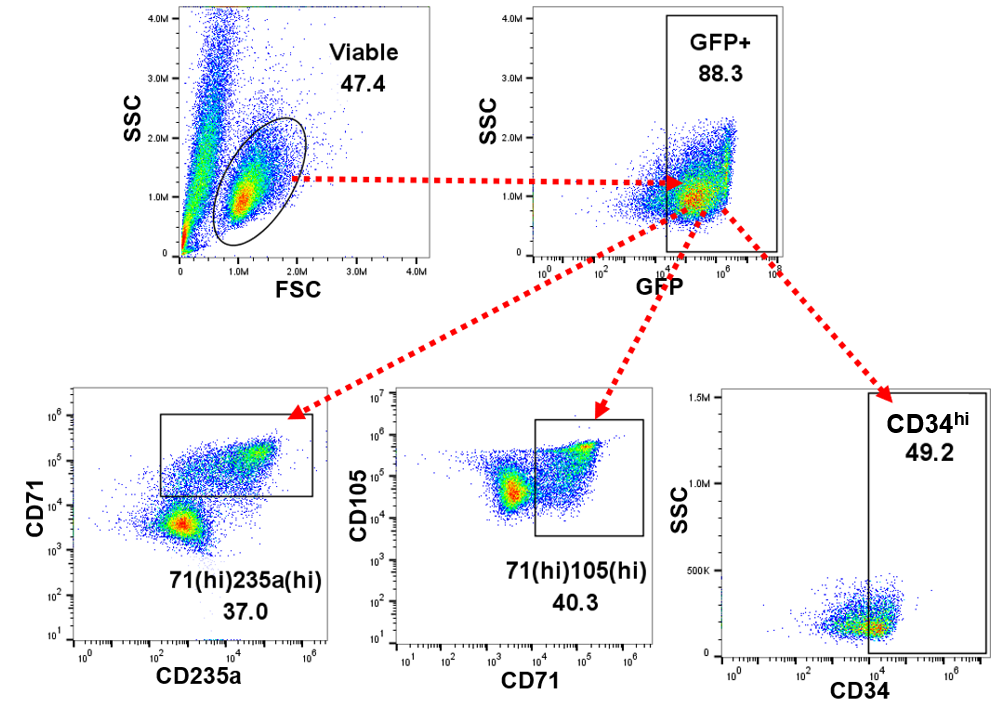


**Supplementary Figure S1. FACS gating strategy for assessing erythroid differentiation in HSPCs.**

On day 3 of erythroid culture, HSPCs transduced with control empty vector (EV) lentivirus were stained with anti-CD71, CD235a, CD34 and CD105 monoclonal antibodies and analyzed by flow cytometry using the Aurora spectral flow cytometer (Cytek). Data was analyzed using FlowJo software (BD Biosciences). In brief, successfully transduced GFP^+^ cell population was gated based on untransduced control cells within the viable cell population, which was defined based on forward scatter (FSC) and side scatter (SSC). Within GFP^+^ cells, the erythroid cell population was gated based on CD71 vs CD235a (CD71^hi^CD235a^hi^) or CD105 vs CD71 (CD71^hi^CD105^hi^) and the stem-progenitor cell population was gated based on SSC vs CD34 (CD34^hi^).


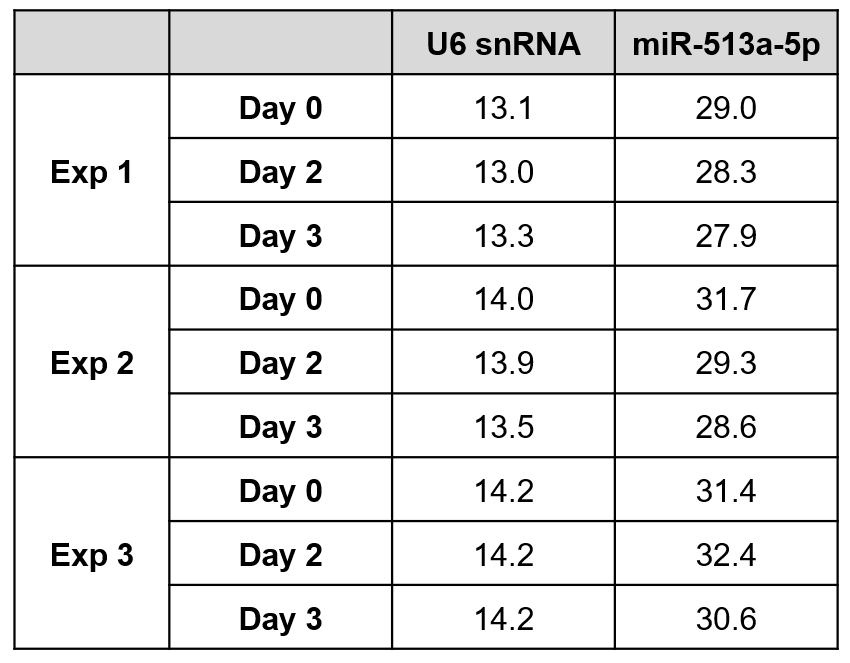


**Supplementary Figure S2. MiR-513a-5p expression increases during early EPO-stimulated erythroid differentiation.** Primary CD34^+^ HSPCs were cultured in EPO-containing media. Total RNA was isolated and quantified on days 0, 2, and 3 of erythroid differentiation culture (n=3). MiR-513a-5p levels were determined by qRT-PCR, and U6 snRNA was used as the endogenous control. The table shows the raw Ct values of U6 snRNA and miR-513-5p for each time point. Within each experiment, U6 snRNA Ct values were similar across days 0, 2, and 3.


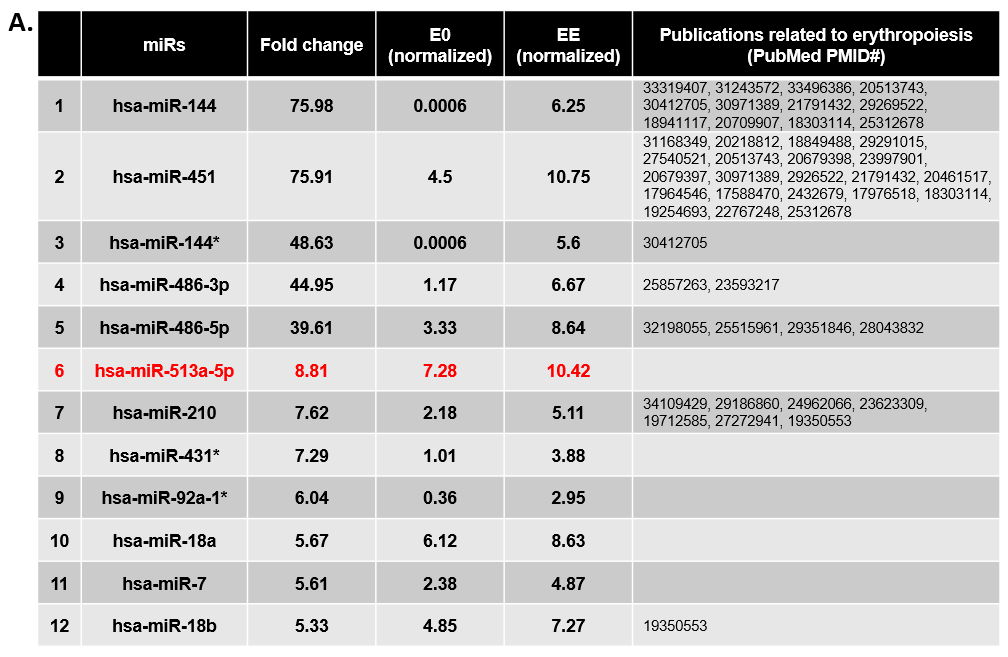


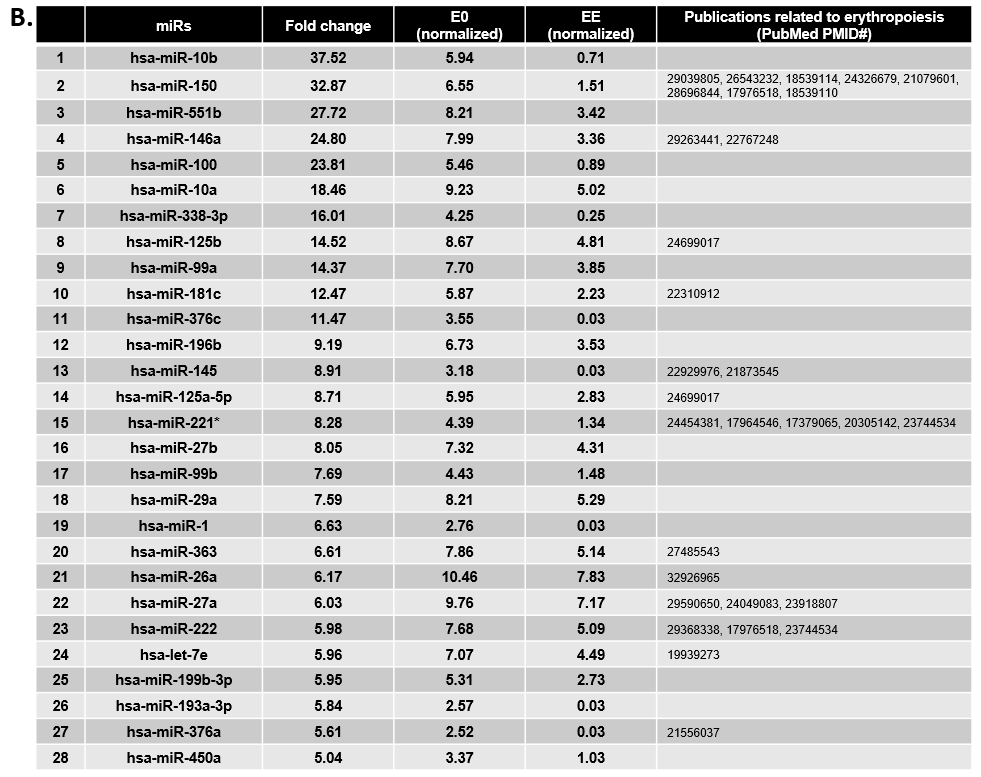


**Supplementary Figure S3. Differentially expressed miRs in early erythroid cells were identified by microarray profiling.** Upregulated or downregulated miRs were selected based on a fold change cutoff of >5. Seven of upregulated miRs (including miR-144, miR-144*, miR-451, miR486-3p, miR-486-5p, mR-210 and miR-18b) and thirteen of downregulated miRs (including miR-150, miR-146a, miR-125b, miR-181c, miR-145, miR-125-5p, miR-221*, miR-363, miR-26a, miR-27a, miR-222, let-7e and miR-376a) have been previously implicated in normal or malignant erythropoiesis. Fold changes and normalized expression values represent averages from two independent miR profiling experiments. The complete list of miRs and mRNAs identified from this profiling experiment is provided in a separately attached Excel file (Supplementary Table S5).

**(A)** List of upregulated miRs in early erythroid cells (EE) compared to uncultured HSPCs (E0).

**(B)** List of downregulated miRs in early erythroid cells (EE) compared to uncultured HSPCs (E0).


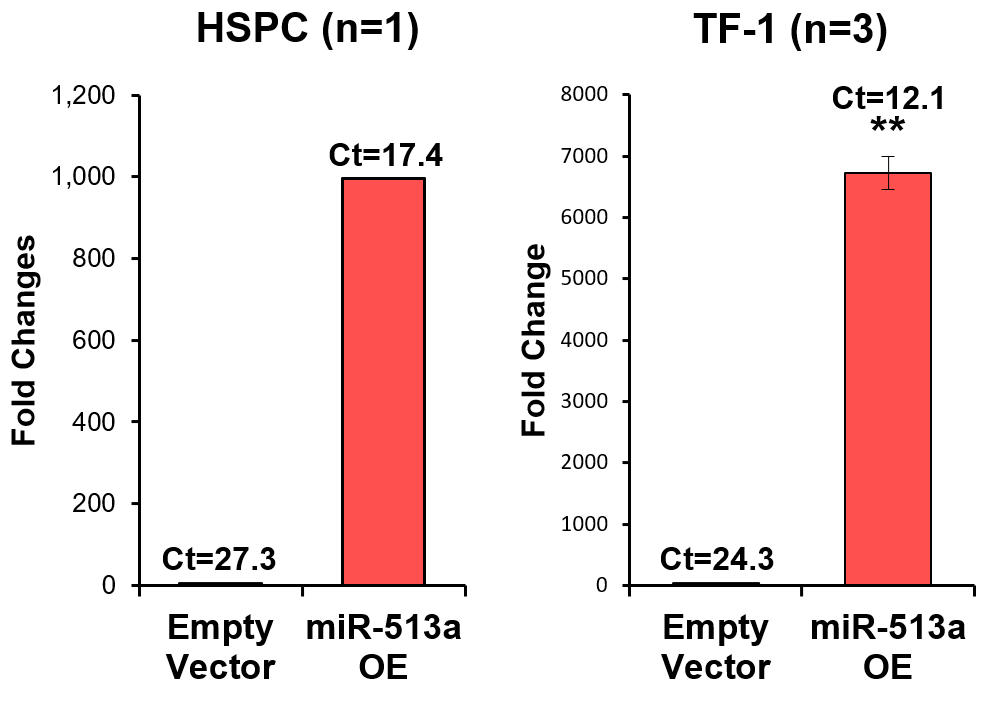


**Supplementary Figure S4. Increased miR-513a-5p expression in miR-513a OE-HSPCs and miR-513a OE-TF-1 cells confirmed by qRT-PCR. (HSPC)** On day 4 of erythroid culture, total RNA was isolated from empty vector control-HSPCs or miR-513a OE-HSPCs. Expression levels of miR-513a-5p were determined by qRT-PCR and U6 small nuclear RNA expression was used as an endogenous control (n=1). **(TF-1)** On day 3 of erythroid culture, total RNA was isolated from empty vector control-TF-1 cells or miR-513a OE-TF-1 cells. Expression levels of miR-513a-5p were determined by qRT-PCR and U6 small nuclear RNA expression was used as an endogenous control (Mean ± SEM, n=3). Statistical significance was determined by Student’s *t*-test (**p<0.005).


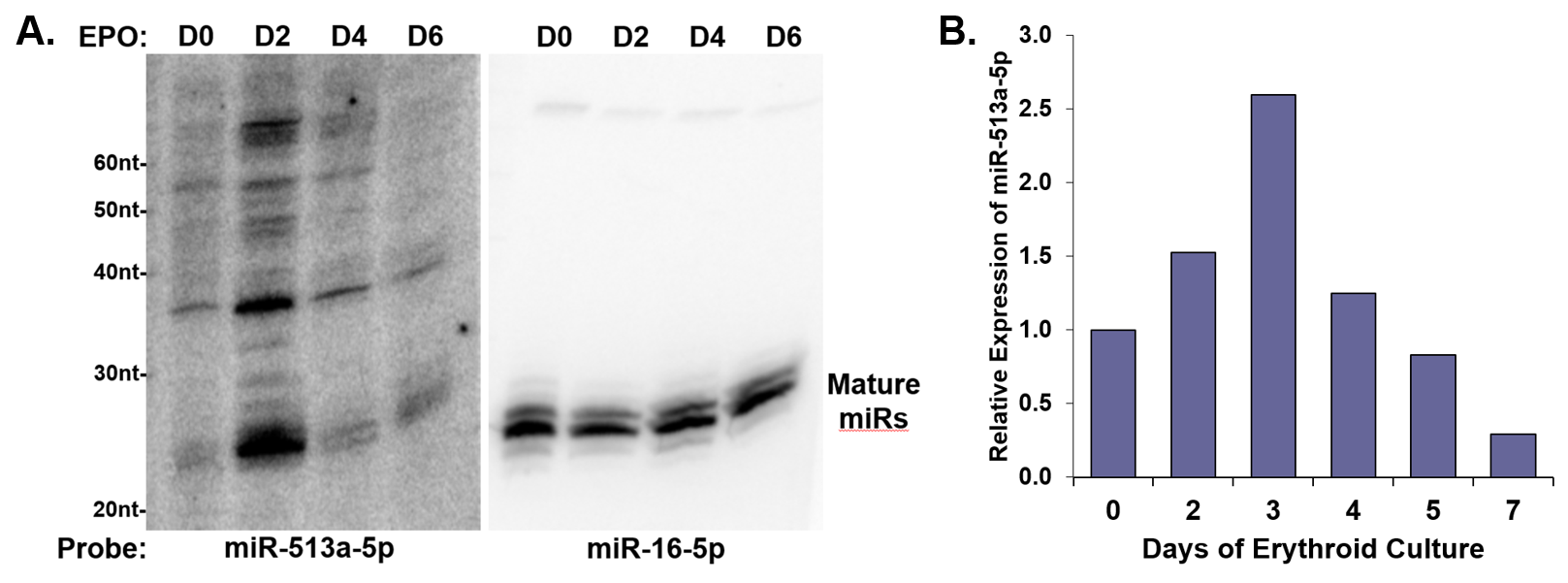


**Supplementary Figure S5. MiR-513a-5p increases early during EPO-stimulated erythroid differentiation in TF-1 cells.**

**(A)** Small RNAs were isolated from TF-1 cells cultured in EPO-containing media on day 0, 2, 4, or 6 using mirVana miRNA isolation kit (Thermo Fisher Scientific). Details of miR Northern blot was previously described with some modification (Yang A. *et al.*, *Mol Cell*, 2019 Aug 8;75(3);511-522; PubMed PMID: 31178353). In brief, 1.5ug small RNA for each sample was separated on a 20% (w/v) acrylamide/8M urea gel, and EDC (1-ethyl-3-(30dimethylaminopropyl) carbodiimide) chemical was used to crosslink RNA to the membrane followed by transferring. Northern blot analysis showed that miR-513a-5p expression increased early during EPO-stimulated erythropoiesis. Endogenous miR-16-5p was used as a loading control (n=1).

**(B)** Total RNAs were isolated from TF-1 cells cultured in EPO-containing media on day 0, 2, 3, 4, 5 or 7. Expression levels of miR-513a-5p were determined by qRT-PCR and U6 small nuclear RNA expression was used as an endogenous control. The bar graph shows the relative expression of miR-513a-5p on each day, as compared to day 0 (n=1).


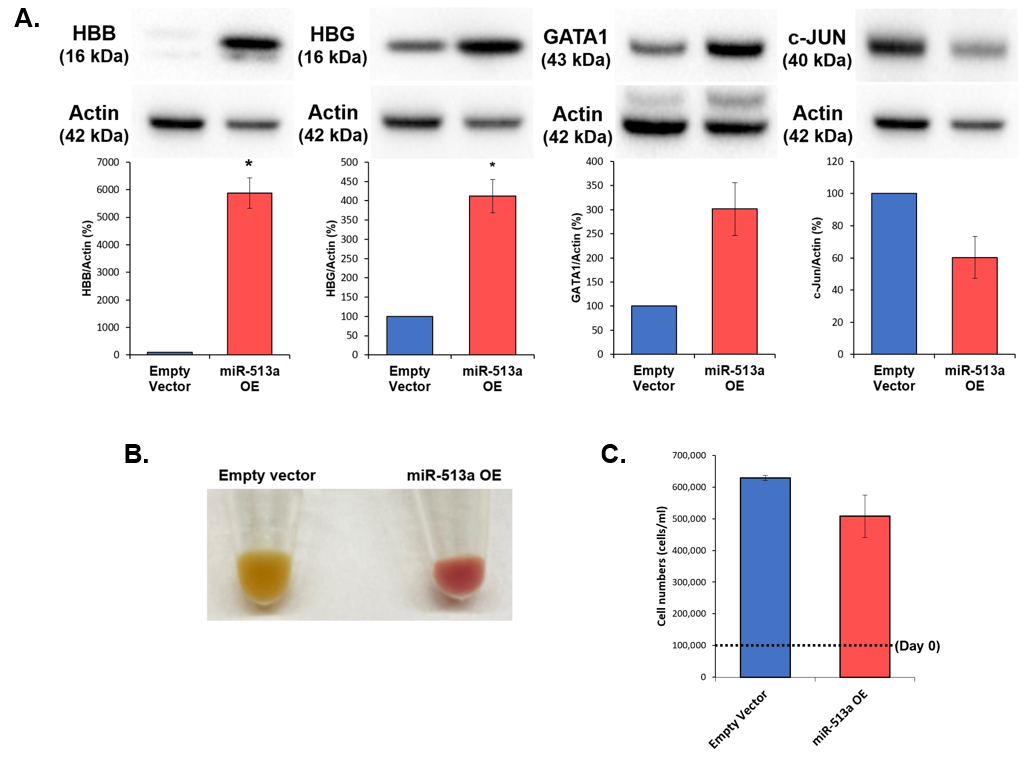


**Supplementary Figure S6. Enforced miR-513a expression promotes erythroid differentiation in K562 cells.**

K562 human chronic myelogenous leukemia cells (RRID: CVCL_0004, American Type Culture Collection CCL-243) were cultured in RPMI1640 media containing 10% FBS. K562 cells were transduced with lentivirus at MOI=5 for 3 days, using 8 ng/ml polybrene as a transduction vehicle. The transduction efficiency was determined by flow cytometry analysis of GFP^+^ cells.

**(A-B)** K562 cells transduced with miR empty vector or miR-513a OE lentivirus were cultured in the standard growth media (without chemical inducers) for 4 days.

**(A)** Transduced K562 cells were analyzed by western blots for HBB, HBG, GATA1, c-Jun and β-Actin (endogenous control) protein levels. Western blots are from a representative experiment. Bar graphs show average normalized protein expression levels determined by densitometry from three independent experiments (Mean ± SEM, n=3). Statistical significance was determined by Student’s *t*-test (*p<0.05).

**(B)** Representative cell pellet photo of miR-513a OE-K562 cells cultured in the absence of chemical erythropoiesis inducers (n=3).

**(C)** After 3 days of transduction, transduced K562 cells were plated at 100,000 cells/ml and cultured in the standard growth media for 2 days. The bar graph shows the average viable cell numbers determined by trypan blue dye exclusion assay (Mean ± SEM, n=3). The dashed line indicates starting cell number on day 0.


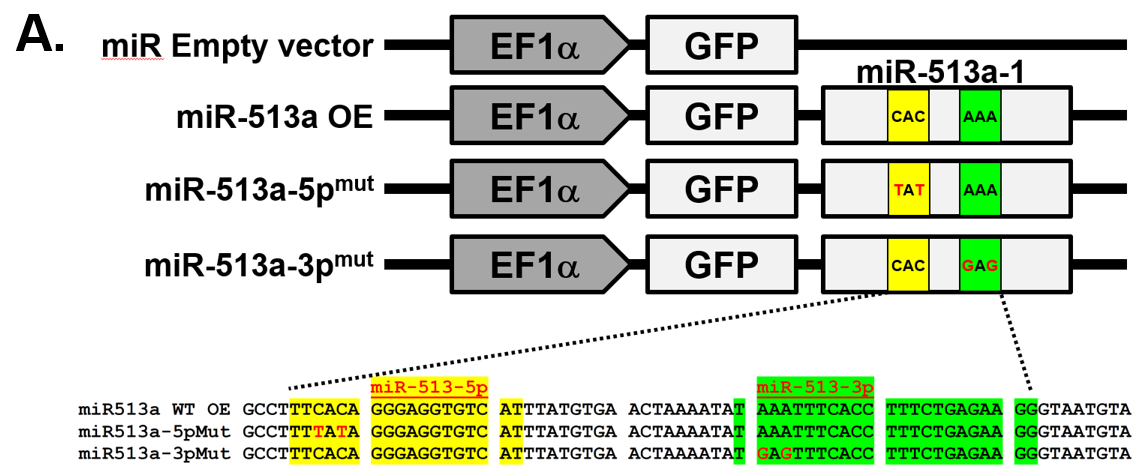


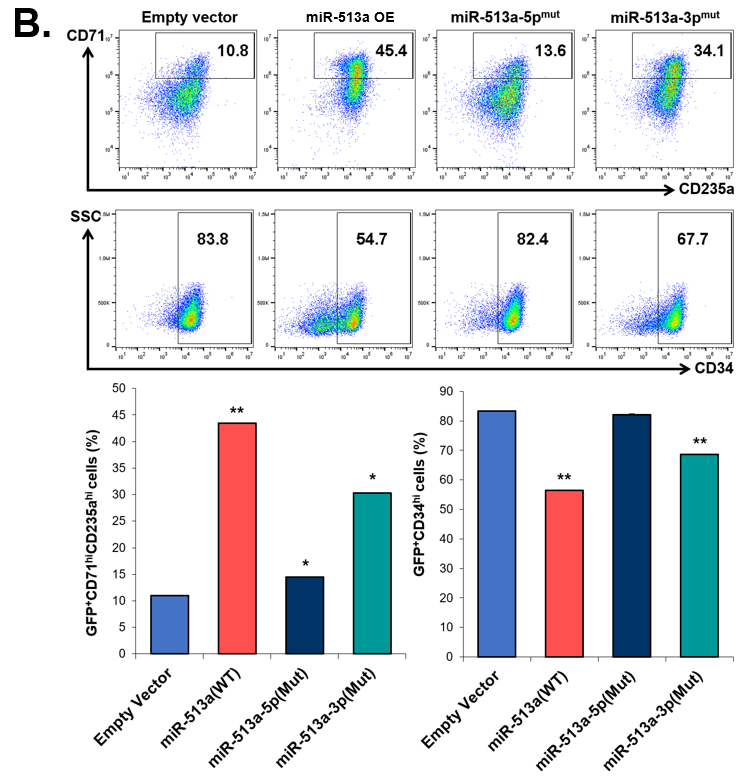


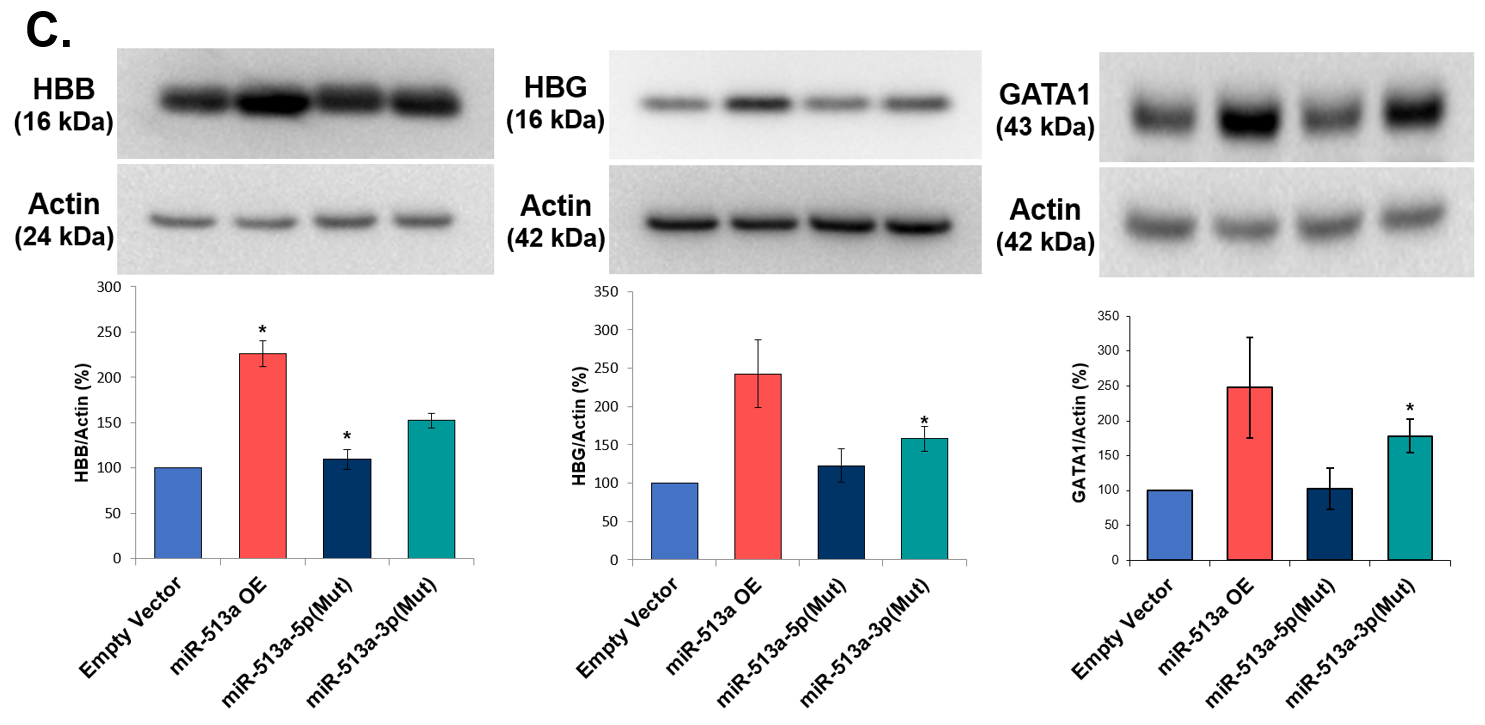


**Supplementary Figure S7. MiR-513a-5p, rather than miR-513-3p, promotes erythroid differentiation in TF-1 cells, in the absence of EPO.**

**(A)** Schematic diagrams and DNA sequence alignment of miR-513a wild type (WT), miR-513a-5p^mut^, or miR-513a-3p^mut^ lentivector. Sequences of mature miR-513a-5p and mature miR-513a-3p are highlighted in yellow and green, respectively. MiR-513a-5p^mut^ or miR-513a-3p^mut^ contains 2-base mutations (in red letters) within the seed sequence of miR-513a-5p or miR-513a-3p.

**(B-C)** TF-1 cells transduced with miR empty vector, miR-513a OE, miR-513a-5p^mut^ or miR-513a-3p^mut^ lentivirus were cultured in the standard GM-CSF media for 2 days.

**(B)** Transduced cells were immunostained with anti-CD71, CD235a, and CD34 antibodies. FACS plots are from a representative experiment. The numbers in the boxes indicate %CD71^hi^CD235a^hi^ (top) or %CD34^hi^ (bottom) in GFP^+^ cells. Bar graphs show average %CD71^hi^CD235a^hi^ (left) or %CD34^hi^ (right) from three independent experiments (Mean ± SEM, n=3). Statistical significance was determined by Student’s *t*-test (*p<0.05, **p<0.005).

**(C)** Transduced cells were analyzed by western blots for HBB, HBG, GATA1 and β-Actin (endogenous control) protein levels. Western blots are from a representative experiment. Bar graphs show average normalized protein expression levels determined by densitometry from three independent experiments (Mean ± SEM, n=3). Statistical significance was determined by Student’s *t*-test (*p<0.05).


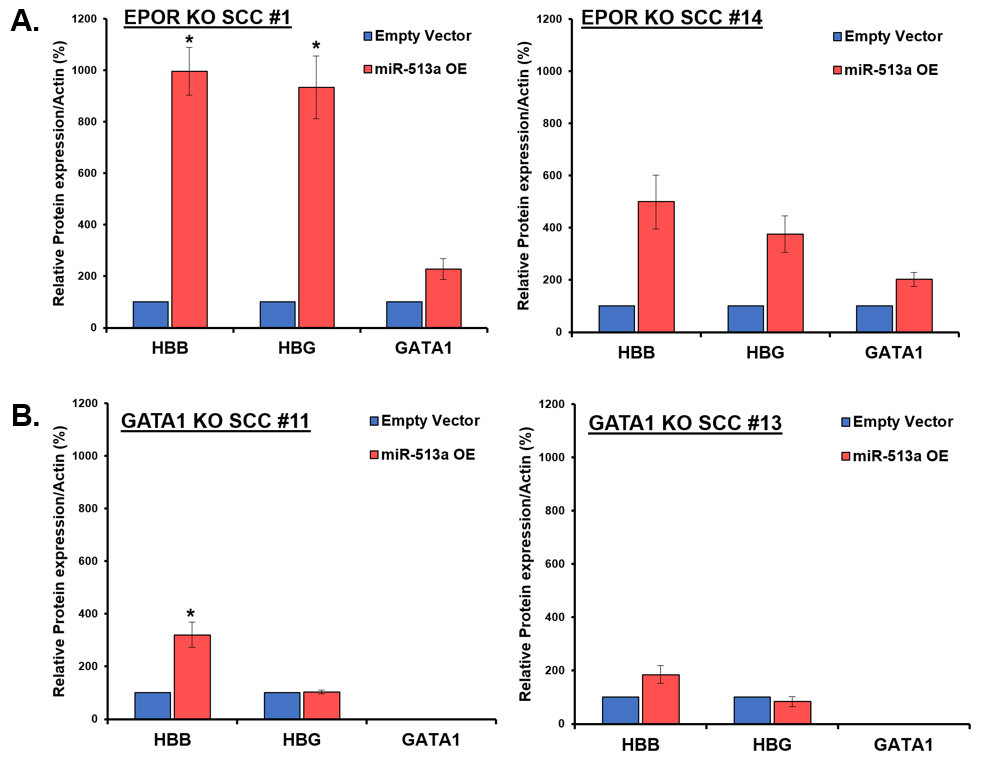


**Supplementary Figure S8. MiR-513a-mediated erythroid differentiation in TF-1 cells requires GATA1 but not EPOR.**

**(A)** EPOR KO TF-1 SCCs (2 clones) or **(B)** GATA1 KO TF-1 SCCs (2 clones) were transduced with miR empty vector or miR-513a OE lentivirus prior to culturing in the standard GM-CSF media (without EPO) for 8 days. Protein expression levels of HBB, HBG, GATA1 and β-Actin (endogenous control) were determined by western blots. Bar graphs show average normalized protein levels determined by densitometry from three independent experiments (Mean ± SEM, n=3). Statistical significance was determined by Student’s *t*-test (*<0.05).


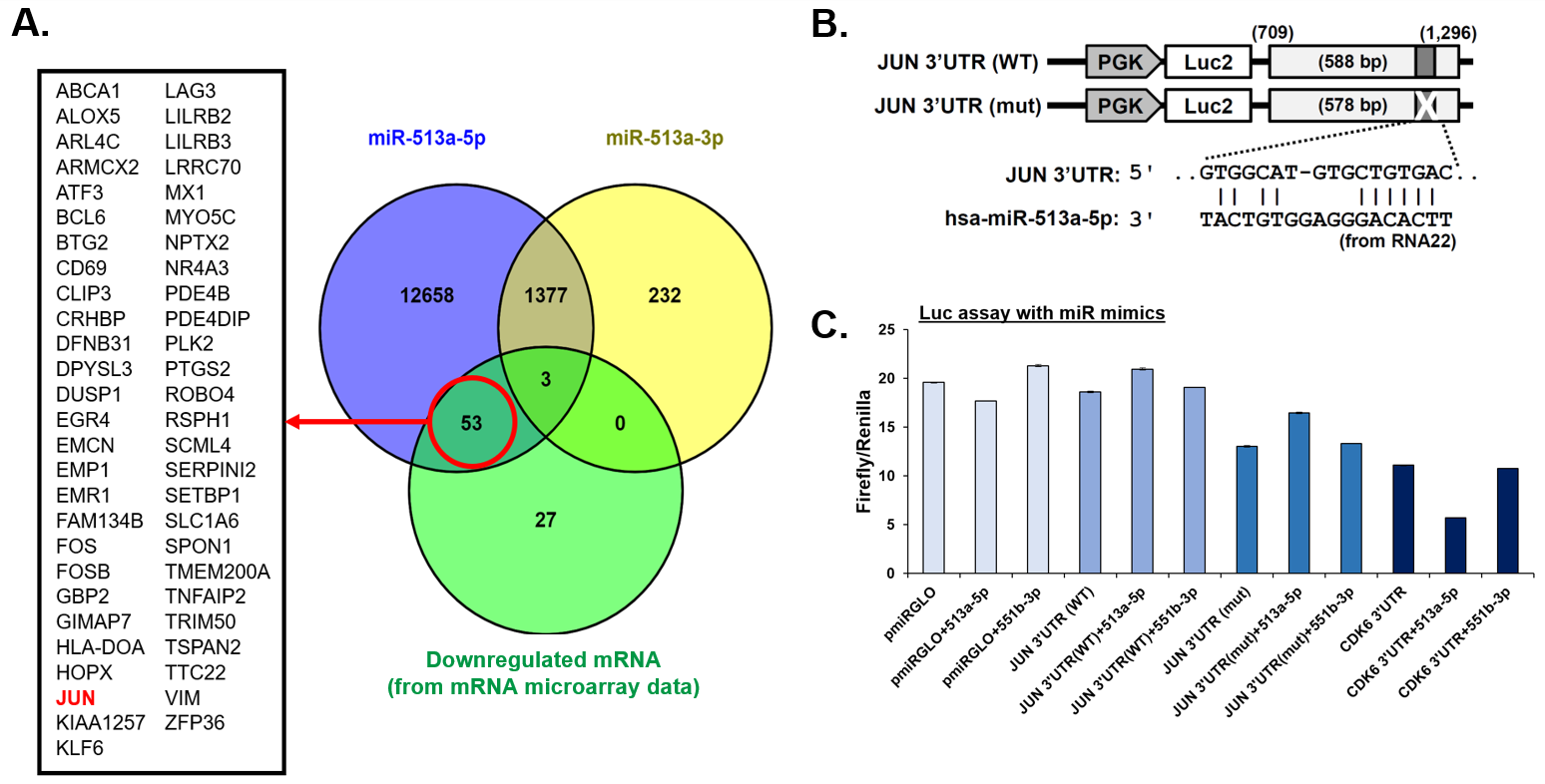


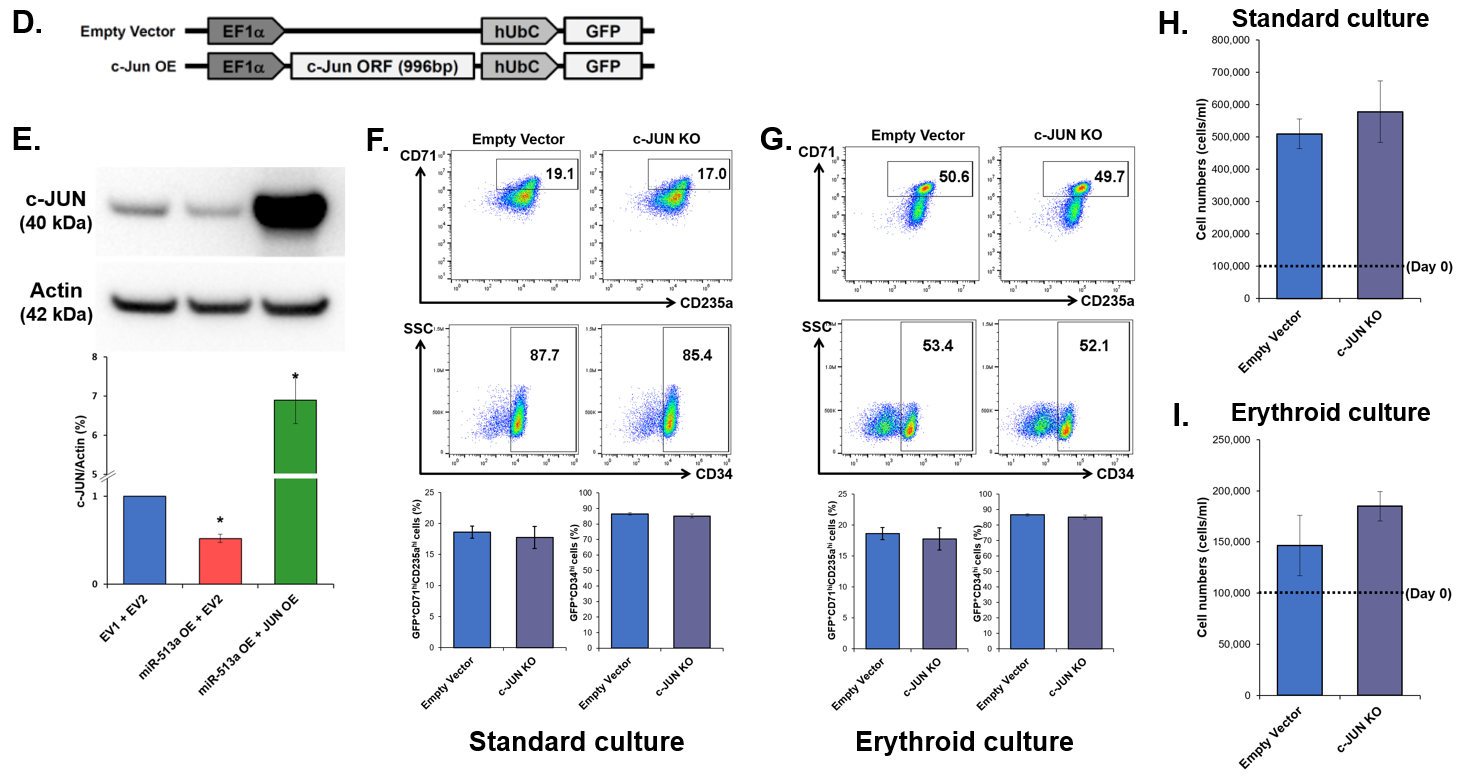


**Supplementary Figure S9. c-JUN is not a direct target of miR-513a-5p, but contributes to miR-513a-mediated erythropoiesis.**

**(A)** Combining bioinformatics (TargetScan; including predicted targets for miR-513a-5p and excluding predicted targets for miR-513a-3p) with our human erythroid gene expression microarray data analysis (list of downregulated mRNAs in EE compared to E0) identified 53 candidate targets of miR-513a-5p. Among these, we focused on c-JUN first as a potential target for miR-513a-5p because c-JUN has previously been reported to be involved in erythropoiesis.

**(B)** Schematic diagram of luciferase constructs with c-JUN 3’UTR wild type (WT) or miR-513a-5p target site deletion mutant. Human c-JUN partial 3’UTR WT (588 bp) and c-JUN 3’UTR deletion mutant (578 bp), each cloned into pTWIST Amp High Copy vector, were purchased from TWIST Bioscience. c-JUN 3’UTR (WT and mutant) sequences, including one predicted miR-513a-5p binding site, were subcloned into pmirGLO Dual-Luciferase miRNA target expression vector (Promega).

**(C)** HEK293T cells were co-transfected with 300 ng c-JUN 3’UTR-luciferase construct (WT or deletion mutant) plus 50 nM miR-513a-5p mimic or miR-551b-3p mimic (unrelated miR control; Thermo Fisher Scientific) using Lipofectamine 2000 (Thermo Fisher Scientific). Transfected HEK293T cells were cultured in DMEM media (Cellgro) containing 10% FBS for 48 hours, and then Firefly and Renilla luciferase activities were measured using the Dual-Luciferase Reporter Assay System (Promega) according to the manufacturer’s instructions on a Victor X3 Multilabel Plate Reader (PerkinElmer). To calculate relative luciferase activity, Firefly luciferase activity was normalized to Renilla luciferase activity. The bar graph shows % relative luciferase activity (n=1). A CDK6 3’UTR wild type (WT) was used as an experimental positive control to confirm miR-513a-5p-mediated repression in the experiment.

**(D)** Schematic diagrams of control empty vector versus c-JUN OE lentivector.

**(E)** c-JUN overexpression in double-transduced miR-513aOE/c-JUN OE-TF-1 cells was confirmed by western blots. Western blots are from a representative experiment. The bar graph shows average normalized protein expression levels determined by densitometry from three independent experiments (Mean ± SEM, n=3). Statistical significance was determined by Student’s *t*-test (*p<0.05).

**(F-I)** CRISPR/Cas9-mediated c-JUN KO TF-1 cells were cultured in standard GM-CSF media for 5 days **(F, H)** or EPO-containing media for 3 days **(G, I)**.

**(F-G)** c-JUN KO TF-1 cells were immunostained with anti-CD71, CD235a, and CD34 antibodies. FACS plots are from a representative experiment. The numbers in the boxes indicate %CD71^hi^CD235a^hi^ (top) or %CD34^hi^ (bottom) in GFP^+^ cells. Bar graphs show average %CD71^hi^CD235a^hi^ (left) or %CD34^hi^ (right) from three independent experiments (Mean ± SEM, n=3).

**(H-I)** Viable cell numbers were counted by the trypan blue dye exclusion assay (Mean ± SEM, n=3). The bar graph shows the average viable cell numbers, and the dashed line indicates starting cell number on day 0.
